# Metagenomic Insights into the Bacterial Functions of a Diesel-Degrading Consortium for the Rhizoremediation of Diesel-Polluted Soil

**DOI:** 10.3390/genes10060456

**Published:** 2019-06-14

**Authors:** Daniel Garrido-Sanz, Miguel Redondo-Nieto, María Guirado, Oscar Pindado Jiménez, Rocío Millán, Marta Martin, Rafael Rivilla

**Affiliations:** 1 Departamento de Biología, Facultad de Ciencias, Universidad Autónoma de Madrid, Darwin 2, 28049 Madrid, Spain; daniel.garrido@uam.es (D.G.-S.); miguel.redondo@uam.es (M.R.-N.); m.martin@uam.es (M.M.); 2 Centro de Investigaciones Energéticas, Medioambientales y Tecnológicas, Avenida Complutense 40, 28040 Madrid, Spain; maria.guirado@ciemat.es (M.G.); oscar.pindado@ciemat.es (O.P.J.); rocio.millan@ciemat.es (R.M.)

**Keywords:** rhizoremediation, diesel, bacteria, consortium, metagenomics, PAHs, TPH

## Abstract

Diesel is a complex pollutant composed of a mixture of aliphatic and aromatic hydrocarbons. Because of this complexity, diesel bioremediation requires multiple microorganisms, which harbor the catabolic pathways to degrade the mixture. By enrichment cultivation of rhizospheric soil from a diesel-polluted site, we have isolated a bacterial consortium that can grow aerobically with diesel and different alkanes and polycyclic aromatic hydrocarbons (PAHs) as the sole carbon and energy source. Microbiome diversity analyses based on 16S rRNA gene showed that the diesel-degrading consortium consists of 76 amplicon sequence variants (ASVs) and it is dominated by *Pseudomonas*, *Aquabacterium*, *Chryseobacterium*, and *Sphingomonadaceae.* Changes in microbiome composition were observed when growing on specific hydrocarbons, reflecting that different populations degrade different hydrocarbons. Shotgun metagenome sequence analysis of the consortium growing on diesel has identified redundant genes encoding enzymes implicated in the initial oxidation of alkanes (AlkB, LadA, CYP450) and a variety of hydroxylating and ring-cleavage dioxygenases involved in aromatic and polyaromatic hydrocarbon degradation. The phylogenetic assignment of these enzymes to specific genera allowed us to model the role of specific populations in the diesel-degrading consortium. Rhizoremediation of diesel-polluted soil microcosms using the consortium, resulted in an important enhancement in the reduction of total petroleum hydrocarbons (TPHs), making it suited for rhizoremediation applications.

## 1. Introduction

Soil pollution by petroleum hydrocarbons, including diesel fuel, is produced by spills and leakages and is a major environmental concern due to the large number of hazardous and toxic constituents [1,2] that lead to reduced germination rates of plant seeds and a decrease in the diversity of the associated soil biota [3,4]. Diesel is a complex mixture of alkanes and polycyclic aromatic hydrocarbons (PAHs) which varies widely depending on the geographical source of the crude oil fraction used during petroleum separation. Furthermore, diesel pollution is usually associated with the presence of heavy metals [5], which also poses an environmental concern due to its toxic effects and produces an acute inhibition of the diesel biodegradation process by microorganisms [6].

Many microorganisms can aerobically degrade alkanes, using them as carbon and energy source [7,8]. Four different pathways for aerobic biodegradation of alkanes have been uncovered to date [7]. Two of these well-studied pathways are initiated by a terminal or subterminal oxidation of a methyl or methylene group, mediated by alkane monooxygenase enzymes, and resulting in the production of primary or secondary alcohols respectively [8,9,10], which are further oxidized by alcohol and aldehyde dehydrogenases into fatty acids before they can enter the beta-oxidation [11]. Alternatively, fatty acids resulting in the alkane terminal oxidation can be further oxidized at the terminal omega methyl group via biterminal oxidation. This process results in the formation of omega-hydroxy fatty acids that are then converted by alcohol and aldehyde dehydrogenases into dicarboxylic acids, which are also funneled into the beta-oxidation [12]. Initial terminal or subterminal oxidation of alkanes is carried out by alkane 1-monooxygenases (AlkB) or long-chain alkane monooxygenases (LadA), which have been extensively characterized [13,14,15,16]. On the other hand, the omega-hydroxy fatty acid formation via the biterminal oxidation pathway is primarily attributed to cytochrome P450 of the PYC153 family [12,17], which can also hydroxylate alkanes on terminal positions to primary alcohols. 

Bacteria can aerobically metabolize PAHs via different well-established pathways [18,19,20]. Metabolism of low molecular weight PAHs, such as naphthalene, anthracene, phenanthrene, and fluorene is usually initiated by the addition of molecular oxygen into the aromatic nucleus mediated by ring-hydroxylating dioxygenases [21,22]. The dihydrodiols formed in this initial step follow a dehydrogenation and then a meta-cleavage mediated by extradiol dioxygenases to give the ring cleavage products, which are further converted into central aromatic intermediates via subsequent series of enzymatic reactions. Many of these PAHs can also be degraded by co-metabolism in the environment [23].

Aged hydrocarbon-polluted soils are characterized by the presence of recalcitrant TPHs, such as branched aliphatic, PAHs and substituted aromatic hydrocarbons, which are usually associated to the organic and clay soil fractions, limiting the access to microorganisms and therefore reducing their biodegradation ability [24,25,26]. In fact, an important factor affecting the bioremediation of hydrocarbon-polluted soils is the presence of appropriate microorganisms [27], along with the soil physicochemical characteristics and other environmental conditions required to support this biota [28]. For bioremediation purposes, the use of indigenous bacterial consortia isolated from the polluted sites, rather than using allochthonous strains, might be advantageous. The effective cooperation of several specialized local microorganisms already adapted to the polluted site in terms of complementary substrate specificity can result in the mineralization of complex hydrocarbon mixtures [29]. Furthermore, as several studies indicate, microbial structure and function are influenced by total petroleum hydrocarbons (TPHs) [30,31]. Therefore, choosing an indigenous population might overcome the problems of bacterial composition shift when introduced into a new environment, which could be replaced by indigenous non-degrading populations that are, however, more competitive. To this purpose, metagenomic approaches have been used to analyze the bacterial composition and its changes [32], and to identify key genes encoding enzymes involved in the pollutant degradation process [33,34]. Rhizoremediation, a type of bioremediation that involves the use of plants to stimulate the activity of petroleum-degrading microorganisms, has been reported to be a cost-effective method for the removal of petroleum hydrocarbons from soil [35,36,37,38]. The combined use of indigenous microorganisms and plants to stimulate their degradation abilities could enhance the removal of hydrocarbons in soil-polluted environments. 

Although most of the studies to date have focused on evaluating the degradation abilities of specific bacterial strains or synthetic bacterial consortia [39], in this work, we report the isolation and characterization of an indigenous soil bacterial consortium that can grow aerobically with diesel oil and other aliphatic and aromatic constituents of diesel as sole carbon and energy source. Metagenomic analysis of the diesel-degrading microbiota allowed us to identify active populations in the degradation of both, aliphatic and aromatic polycyclic hydrocarbons by the assignation of specific key coding DNA sequences (CDSs) to certain genera. Finally, we have tested this consortium in real diesel-polluted soil microcosms to address its potential for rhizoremediation. 

## 2. Materials and Methods

### 2.1. Isolation of the Bacterial Consortium and Growth Conditions

Standard successive enrichment culture procedures were used to isolate the diesel-degrading bacterial consortium. Briefly, samples were collected from the rhizosphere of two plant species: *Tamarix gallica* and *Pistacia lentiscus*, planted in an aged diesel-polluted soil. Pollution came from ship fuel tanks spills in San Fernando (Cádiz, Spain. 36.497624 N, 6.191080 W). 2 g of diesel-polluted rhizospheric soil was added to 500 mL of sterile liquid minimum salt medium (MM) [40], supplemented with 1 mL/L of phosphate-buffered mineral medium salts (PAS) [41] and 0.005% yeast extract, and grown at 28 °C with shaking (140 rpm). One mL/L of diesel oil (from the ship fuel tank) was added as the sole carbon and energy source. After five subcultures, 20 mL aliquots after 48 h of growth were centrifuged for 10 min at 4000× *g*. The pellet was then resuspended in 0.75 mL of MM+PAS and mixed with 0.25 mL of glycerol 80% and deep-frozen at −80 °C. The isolated consortium was routinely grown in a liquid culture of MM+PAS supplemented with 1 mL/L of diesel oil as the sole carbon and energy source and 0.005% yeast extract, at 28 °C with shaking (140 rpm).

The culture growth on different aliphatic and aromatic compounds as sole carbon and energy sources was evaluated as above, but *n*-hexane, *n*-heptadecane, *n*-tetracosane, naphthalene, and phenanthrene (1 mL or 1 g/L) were added as the sole carbon and energy source. In the case of hexane, sterile filter paper was soaked in hexane, added to the flask caps and sealed to prevent its evaporation. 

### 2.2. DNA Extraction, Sequencing, and Assembly

DNA extraction from the bacterial consortium after 48 h of growth (Supplementary Figure S1), on diesel oil, hexane, pentadecane, heptadecane, tetracosane, naphthalene, or phenanthrene as sole carbon and energy source was performed using the Realpure Genomic DNA Extraction Kit (Durviz, Spain). Illumina sequencing of 16S rRNA amplicons in all samples and whole-metagenome shotgun of the consortium growing with diesel as sole carbon source was carried out by Parque Científico de Madrid (Spain). Briefly, the 16S rRNA genes in each sample were sequenced by means of amplification of the V3-V4 16S rRNA region with the primers 16SV3-V4-CS1 (5′-CCT ACG GGN GGC WGC AG-30) and 16SV3-V4-CS2 (5′-GAC TAC HVG GGT ATC TAA TCC-3′), position 341 to 785 in *Escherichia coli*, prior to libraries preparation with Illumina MiSeq v3 reagent kit according to supplier specifications, and sequenced by Illumina MiSeq paired 300-bp platform. Whole-metagenome of the diesel-growing bacterial consortium was sequenced using Illumina TruSeq preparation kit, a mean library size of 483 bp and illumina MiSeq paired 300-pb.

Raw reads were trimmed and quality-filtered using Trimmomatic v0.36 software [42] to remove those with less than 50 nts in the case of microbiomes or 100 nts in the metagenome, resulting in a read recovery rate ranging from 95.9% to 97.5% in the microbiomes and 97.2% in the metagenome reads. Trimmed reads from the metagenome sequencing were assembled using SPAdes v3.12 software [43], metaSPAdes option, and default settings. Assembly quality was evaluated using QUAST v4.4 [44]. The resulting contigs were annotated using the RAST pipeline [45].

### 2.3. Diversity Analysis of the 16S rRNA Gene and Coding DNA Sequences (CDSs)

Microbiome 16S rRNA gene diversity was assessed with QIIME v2-2019.4 [46]. Briefly, cleaned and trimmed paired reads (described above) were filtered and denoised using DADA2 [47]. For chimera identification, 200,000 training sequences were used. Identified amplicon sequence variants (ASVs) were aligned using MAFFT [48] and further processed to construct a phylogeny with fasttree2 [49]. Rarefaction curves and Shannon Index were estimated using the plugin q2-diversity running 10 iterations, and 1000 sequence steps up to the maximum number of sequences per sample. Taxonomy was assigned to ASVs using the q2-feature-classifier [50], classify-sklearn naïve Bayes taxonomy classifier against the SILVA v132 99% 16S sequence database [51]. A specific classifier for the amplified 16S region was trained using the primers specified above and a maximum fragment size of 300 nts. 

To assess the diversity of coding DNA sequences (CDSs), after whole-metagenome assembly and annotation (as specified above), CDSs were searched against the NCBI nucleotide (nt) database (October 2018) using blastn from BLAST v2.2.31+ software [52]. For each query, the first hit with a minimum of 75% sequence identity and 50% coverage was used for genus assignation.

### 2.4. Identification of CDSs Involved in Alkanes and Aromatic Hydrocarbons Metabolism

Coding DNA sequences of alkane 1-monooxygenase (AlkB), long-chain alkane monooxygenase (LadA), cytochrome P450 alkane hydrolase (CYP153 family) and extra and intradiol ring-cleavage and ring-hydroxylating dioxygenases (catechol 2,3-dioxygenase, biphenyl-2,3-dioxygenase, 3-carboxyethylcatechol 2,3-dioxygenase, 3-hydroxyantranilate 3,4-dioxygenase, 3-*O*-methylgallate 3,4-dioxygenase, 2,3-dihydryphenylpropionate 1,2-dioxygenase, 4,5-DOPA dioxygenase, 2-aminophenyl-1,6-dioxygenase, protocatechuate 4,5-dioxygenase, 3,4-dihydroxyphenylacetate 2,3-dioxtygenase, catechol 1,2-dioxygenase, protocatechuate 3,4-dioxygenase, gentisate 1,2-dioxygenase, homogentisase 1,2-dioxygenase, anthranilate 1,2-dioxygenase, benzoate 1,2-dioxygenase, naphthalene 1,2-dioxygenase, 2-halobenzoate 1,2-dioxygenase, biphenyl 2,3-dioxygenase, 3-phenylpropanoate dioxygenase and p-cumate 2,3-dioxygenase) were identified in the diesel oil-degrading bacterial metagenome by means of annotations and validated by blast searches against the nucleotide (nt) NCBI database (October 2018). Results were further filtered by 75% sequence identity, 50% coverage and a minimum of 1 × 10^−10^ expected value. For queries without significant hits against nt NCBI database, protein searches against non-redundant (nr) NCBI database (October 2018) were used instead. 

### 2.5. Bioremediation Treatments in Microcosms

To evaluate the bioremediation feasibility of the diesel-degrading bacterial consortium, four-month microcosms systems with two different treatments were evaluated. The microcosms and the treatments are detailed below: 

**(a) Soil homogenization processing.** Diesel-polluted bulk soil from ship fuel tank spills was collected in San Fernando (Cádiz, Spain). The soil was homogenized by a first sieving process with a < 4 mm net, followed by manual homogenization. The soil was then automatically quartered with 2, 4 and 8 divisions. Finally, 200 g of this sieved, homogenized and quartered soil was included in an automatic tumbler for 12 h to ensure homogeneity before placing it into pots. The initial diesel concentration of the pot’s soil was 2974 ± 143 mg·kg^−1^. The soil had a water holding capacity of 32.25 mL·100 g^−1^, a pH of 8.165, an electrical conductivity of 203 µL·cm^−1^, 512.1 mg·L^−1^ of nitrogen, 4.32 mg·kg^−1^ of phosphate and 23.16 mg·kg^−1^ of easily oxidizable carbon (EOC).

**(b) Treatment 1.** Pots with 200 g of the homogenized soil previously described were surface-inoculated once, at the beginning of the experiment, with 1 mL of washed diesel-degrading bacterial consortium after 48 h of growth and concentrated to a final DO_600_ = 0.6. Four replicates of the treatment 1 together with other four control replicates without the bacterial inoculum were placed.

**(c) Treatment 2.** Five one-week old alfalfa (*Medicago sativa*) were transplanted into each pot, consisting in 200 g of the homogenized soil previously described. Alfalfa seeds were surface sterilized with 70% ethanol for 3 min and 5% NaClO for 10 min, washed 10 times with sterile distilled water and pre-germinated in 1% (w/v) sterilized agar-water plates at 28 °C before transplant. 1 mL of the bacterial consortium specified above was inoculated per pot to the stem base of the plants (0.2 mL per plant). Four replicates of the treatment 2 together with other four control replicates without the bacterial inoculum were placed.

**(d) Microcosms conditions.** The two treatments together with the controls were kept for four months in culture chambers with a photoperiod of 16/8 h light/dark and 25/18 °C and maintaining an 80% soil humidity with Fahraeus Plant (FP) medium [53] when needed. The experiment started when the bacterial inoculum was added.

### 2.6. Total Petroleum Hydrocarbon and PAHs Characterization

Total petroleum hydrocarbons (TPHs) and other hydrocarbon fractions in soils, were analyzed by gas chromatography (GC) according to the procedure previously described [54]. Briefly, 1 g of duplicates dry soil samples were microwave-extracted by a mixture of hexane/acetone (1:1) and extracts with petroleum hydrocarbons were subsequently fractioned by a solid phase extraction (SPE) procedure. Aliphatic and aromatic fractions were finally analyzed by GC with a flame ionization detector (GC-FID). Sample analyses for PAH determination in the diesel fuel used for enrichment cultures were performed on an Agilent series 1200 high-performance liquid chromatograph (HPLC) coupled to an Agilent 1100 fluorescent detector (FD, Waldbronn, Germany). Diesel was weighted to obtain more precise PAHs measurements. Particular conditions were previously optimized [55].

### 2.7. Sequence Deposition

Raw reads of the microbiomes 16S rRNA gene amplicons and the whole-metagenome shotgun sequence of the diesel-degrading consortium have been deposited in the NCBI Sequence Read Archive (SRA) and are available under the BioProject accession number PRJNA525339 and SRAs SRR8663212-SRR8663218. 

## 3. Results and Discussion

### 3.1. Diesel Characterization

The initial characterization of the aliphatic and aromatic hydrocarbon fractions in the diesel oil from ship fuel tanks and the aged diesel-polluted soil used in this study by means of gas chromatography [54], shows a prevalence of middle-chain to long-chain aliphatic hydrocarbons (C_12_ to C_35_) and C_16_–C_35_ aromatic hydrocarbons (Table 1). The aged soil, compared with the diesel oil from the tanks, is enriched in aliphatic >C_21_–C_35_ and >C_35_ and aromatic >EC_21_–EC_35_ and >EC_35_ fractions while a reduction in aliphatic >C_10_–C_12_ in the diesel-polluted soil is observed. This was expected as short-chain alkanes are more volatile and prone to bioremediation than long-chain alkanes and PAHs [56]. 

Among the aromatic hydrocarbon fraction of the diesel oil analyzed by HPLC/FD, the composition of the diesel oil is mainly supported by 2-methylnaphthalene (4000 µg·g^−1^), 1-methylnaphthalene (1300 µg·g^−1^), and naphthalene (870 µg·g^−1^). Other constituents are phenanthrene (720 µg·g^−1^), fluorene (230 µg·g^−1^), acenaphthene (90 µg·g^−1^), pyrene (40 µg·g^−1^), anthracene (30 µg·g^−1^), chrysene (27 µg·g^−1^), benzo(b)fluoranthene (2.6 µg·g^−1^), benzo(k)fluoranthene (2 µg·g^−1^), and benzo(a)pyrene (2 µg·g^−1^). Trace aromatic hydrocarbons are fluoranthene (<1 µg·g^−1^), benzo(a)anthracene (<1 µg·g^−1^), dibenzo(ah)anthracene (<1 µg·g^−1^), and benzo(ghi)perylene (<1 µg·g^−1^).

### 3.2. Bacterial Diversity in the Diesel-Degrading Consortium

**Figure 1:**
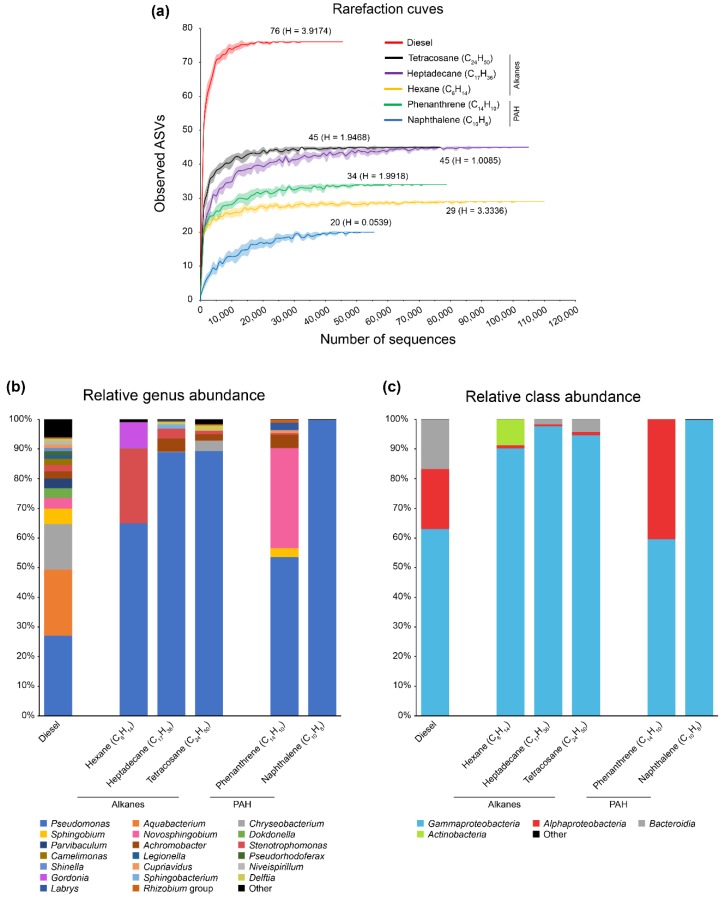
Diversity and taxonomic composition of the diesel-degrading consortium growing on diesel and different alkanes and polycyclic aromatic hydrocarbons (PAHs) as sole carbon and energy source. (**a**) Rarefaction curves of observed amplicon sequence variants (ASVs) over the number of sequences sampled. Lines represent mean values while colored shadows represent standard deviation over 10 iterations. All curves show complete community coverages. Number of ASVs in each microbiome and Shannon diversity index (H) are indicated above/below each curve. (**b**) Relative genus abundance or (**c**) class abundance based on 16S rRNA. Only taxa with a minimum relative abundance of 1% across samples are represented. For detailed abundance distribution, see Supplementary Table S1.

Sequencing of the 16S rRNA gene in the diesel-degrading consortium resulted in a total of 47,306 sequences assigned to 76 different amplicon sequence variants (ASV). The rarefaction curve obtained (Figure 1a) shows a clear community coverage, as saturation of observed ASVs is achieved before 40,000 sequences and the presence of other taxa in the consortium is unlikely. The relative abundance of genera assigned to these sequences shows dominance of *Pseudomonas*, *Aquabacterium*, and *Chryseobacterium*, with relative abundances of 27.01%, 22.36%, and 15.34%, respectively (Figure 1b). Other genera with a representative abundance in the diesel-degrading consortium are *Sphingobium*, *Novosphingobium*, *Dokdonella*, *Parvibaculum*, and *Achromobacter* (5.2%, 3.65%, 3.29%, 3.24%, and 2.45%% of relative abundance, respectively). This abundance is detailed in Supplementary Table S1. On the other hand, the metagenome shotgun sequencing of the diesel-degrading consortium resulted in 140 Mbps, distributed in 114,357 contigs (Supplementary Table S2), 18,473 of them > 1 Kbp. After annotation, 120,867 CDSs were identified and roughly 65% of them could be assigned to the genus level (78,110). The relative abundance of these CDSs in the diesel-degrading consortium shows a major difference of populations, as shown in Supplementary Table S1. Although CDSs of *Pseudomonas* remain as the most abundant (15.53%), CDSs of *Aquabacterium* and *Chryseobacterium* are scarce with relative abundances of 0.04% and 2.82%, respectively, while CDSs from *Achromobacter* and *Commamonadaceae* bacteria are increased (11.07% and 15.08% in CDSs abundance, while 2.45% and 2.64% in 16S rRNA abundance, Supplementary Table S1). Interestingly, other genera that have little representation in 16S rRNA sequences appear in the CDSs genus assignation, such as *Cupriavidus* (6.99% in CDSs while 1.15% in 16S rRNA). However, these differences between 16S rRNA and CDSs are diminished at the class level (Supplementary Table S1), which might suggest an unreliable genus assignation of CDSs, lack of representative sequences for all genera in the NCBI nt database, a primer bias of 16S rRNA sequence or failed prediction of ORFs in small contigs. This result was not unexpected, as it has been previously reported [33]. The taxa identified in the microbiome of the diesel-degrading consortium are in agreement with previous works, where it has been shown what *Pseudomonas* is one of the most abundant genera on hydrocarbon-polluted soils [34,57,58] and *Aquabacterium* and *Chryseobacterium* are also common members in hydrocarbon-degrading bacterial communities [58,59]. Although *Pseudomonas* also rules the degradation of PAHs in sediments [60], other genera present in the diesel-degrading consortium belonging to the *Sphingomonadaceae* family have also been previously reported to be responsible for the degradation of different PAHs [61,62].

### 3.3. Substrate-Specific Diversity

To address the changes in populations of the diesel-degrading consortium that might be occurring due to specific constituents of diesel, microbiome analyses were performed with the consortium growing on three different *n*-alkanes (hexane, heptadecane, and tetracosane) and two PAHs (phenanthrene and naphthalene) as sole carbon and energy source. It is important to note that growth patterns and yield were different on different hydrocarbons (Supplementary Figure S1). The yield was very low in the case of phenanthrene. When growing on this hydrocarbon, OD_600_ at sampling time (48 h) was only 0.06, compared to 0.25–0.5 for the other hydrocarbons. Therefore, phenanthrene is a poor carbon and energy source for bacteria present in the consortium. Regarding the growth pattern, polyphasic curves were obtained for growth on hexane, heptadecane, tetracosane, and phenanthrene, indicating probably a succession in the bacterial populations present in the consortium. Taken together, it is likely that depending on the carbon and energy source, different populations are thriving at different times, and therefore, the detected microbiota reflects a snapshot at the sampling time. As expected, the number of ASVs varies greatly depending on the specific substrate, as shown in Figure 1a. While the consortium growing in tetracosane and heptadecane presents the highest number of ASVs (45), followed by phenanthrene (34), hexane (29), and naphthalene (20), the Shannon diversity index is considerably higher in the consortium growing with hexane (H = 3.3) and lower in the consortium growing with naphthalene (H = 0.05) (Figure 1a). Rarefaction curves, in all cases show nearly complete community coverage. Regarding bacterial abundance, all alkanes are dominated by *Pseudomonas*. In the case of heptadecane and tetracosane, *Pseudomonas* represent the ~89% of the bacterial community and little changes are observed in the remaining genera, none of them representing more than 5% of relative abundance (Figure 1b, Supplementary Table S1). The similarity of the bacterial populations in both, middle and long-chain alkanes, suggest that the same populations are involved in the degradation of both hydrocarbons. On the other hand, diversity of the consortium growing with hexane, shows a dominancy of *Pseudomonas* (64.92%), *Stenotrophomonas* (25.23%), and *Gordonia* (8.7%). *Gordonia* is known to degrade short-chain gaseous alkanes, such as propane [10,63], which could explain the abundance of this genera in the consortium growing with hexane, although there are also reports of *Pseudomonas* strains that are also able to degrade short-chain alkanes [64]. In the case of *Stenotrophomonas*, it has been suggested that the high metabolic versatility of this genus [65] might contribute to its ubiquity in different bacterial populations, including hydrocarbon and PAH-degrading communities [33,60,66] by cross-feeding on secondary metabolites. 

Regarding the relative abundance of the consortium growing with two different PAHs as sole carbon and energy source, naphthalene-degrading diversity is almost exclusive to *Pseudomonas*, representing the 99.72% of the bacterial community (Figure 1b). On the other hand, the bacterial populations that thrive in the phenanthrene culture are mainly distributed between *Pseudomonas* (53.6%) and *Novosphingobium* (33.69%), which is in agreement with previous reports [60,61,62]. 

### 3.4. Identification of Alkane-Degrading CDSs

**Figure 2:**
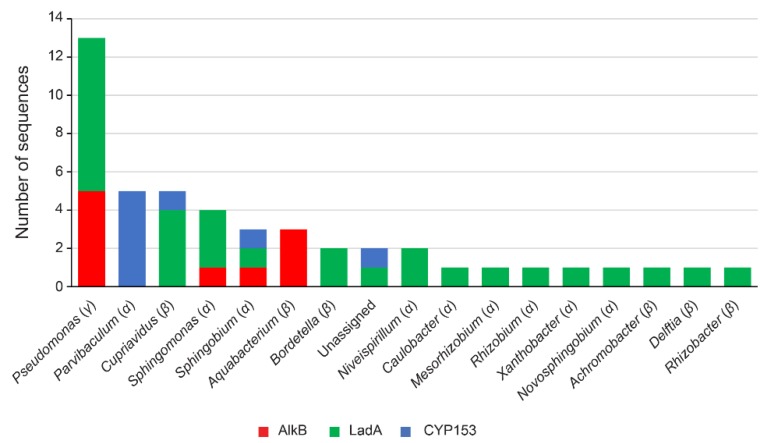
Number and taxonomic assignation at the genus level of the enzymes belonging to alkyl group hydroxylases; AlkB (alkane 1-monooxygenase), LadA (long-chain alkane monooxygenase), and CYP153 (cytochrome P450 family CYP153). Class adscription of the genera depicted is indicated under parenthesis. For additional information, see Supplementary Table S3.

In order to identify putative active populations in the degradation of alkanes, the metagenome CDSs of the diesel-degrading consortium were screened to find alkane 1-monooxygenases (AlkB), cytochrome P450 alkane hydroxylases from the CYP153 family and long-chain alkane monooxygenases (LadA), whose role in *n*-alkane degradation have been extensively studied [7,16,17,67,68]. The results are summarized in Figure 2 (for details see Supplementary Table S3).

AlkB is a non-heme iron integral membrane protein that is responsible for the initial hydroxylation of a diverse range of *n*-alkanes [8,13,67]. Ten putative AlkB have been identified in the metagenome of the diesel-growing bacterial consortium. Half of these AlkB have been classified as belonging to the *Pseudomonas* genus (5), three were assigned to *Aquabacterium*, and the remaining ones were classified as belonging to *Sphingomonas* and *Sphingobium* (Figure 2). On the other hand, LadA, a flavoprotein monooxygenase that inserts an oxygen atom into long-chain alkanes [16], was putatively found 29 times in the metagenome and was mainly assigned to *Pseudomonas* (8), *Cupriavidus* (4) and *Sphingomonas* (3) among others (Figure 2). Finally, CYP153 family of cytochrome P450 have been reported to display hydroxylating activity toward alkanes [17,68]. The metagenome of the diesel-growing consortium contains eight of these enzymes, which have been classified as belonging to *Parvibaculum* (5), *Sphingobium* (1), and *Cupriavidus* (1), while the remaining one could not be assigned to any genera.

These results are in agreement with the relative abundance of *Pseudomonas*, *Aquabacterium,* and *Sphingobium,* and other *Sphingomonadaceae* genera in the diesel-growing consortium and suggest that different genera are active in the degradation of the alkane constituents of diesel. The fact that the consortium growing on heptadecane and tetracosane is dominated by *Pseudomonas* could indicate that the rest of AlkB, LadA, and CYP153-containing bacteria plays a predominant role in the degradation of other alkanes or are specific for a certain alkane length or pathway. For instance, CYP153 coding sequences, which have been primarily assigned to *Parvivaculum* suggest that biterminal oxidation of alkanes is specific to this genus, although it could also hydroxylate alkanes on terminal positions. On the other hand, the diversity of ASVs found in the consortium growing with the long-chain alkane tetracosane (Figure 1) is congruent with the number of LadA enzymes found in the metagenome (29), although AlkB could also be involved on long-chain alkane degradation [15]. It is important to note that also genes participating in the early oxidation of alkanes or those belonging to low abundant bacteria could be missing from the analysis given that metagenome analysis was performed after 48 h of the diesel-degrading consortium growth.

### 3.5. Identification of PAH-Degrading CDSs and Central Aromatic Metabolism CDSs

**Figure 3:**
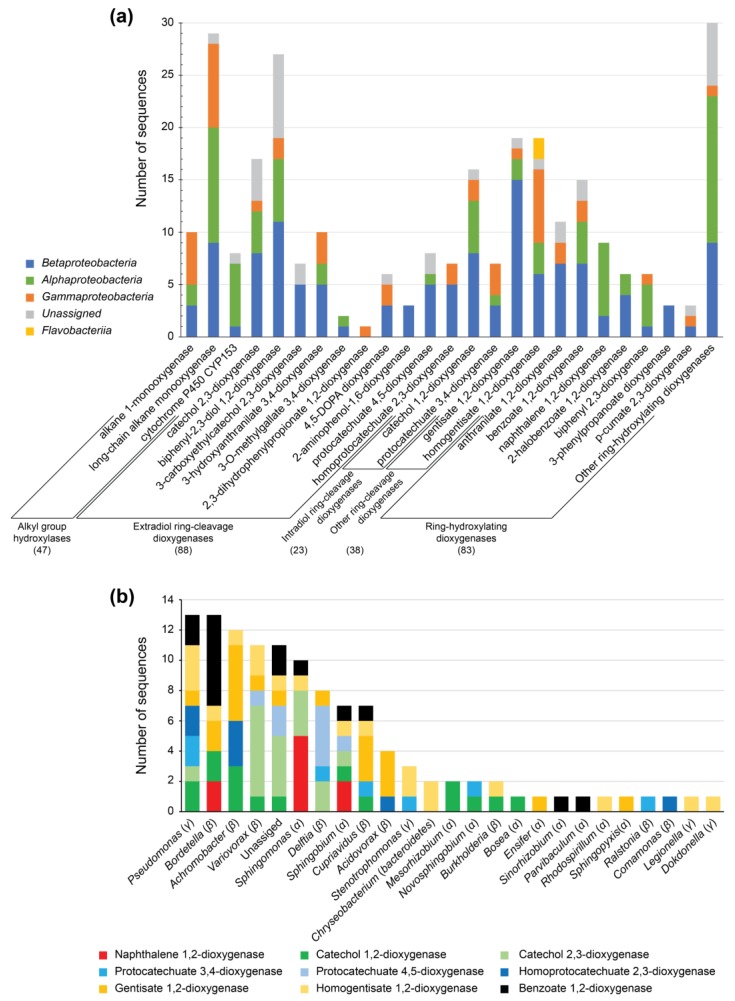
(**a**) Number and taxonomic assignation at the class level of the enzymes belonging to alkanes, PAHs and aromatic metabolism pathways. Total sequences of each main protein groups are indicated in parenthesis. (**b**) Classification at the genus level of naphthalene 1,2-dioxygenase and other central aromatic biodegradation pathways involving catechol, protocatechuate, gentisate, and benzoate. For additional information, see Supplementary Table S3.

Among PAHs present in diesel oil, naphthalene and its methyl derivatives are the most abundant (see above). Naphthalene biodegradation is initiated by the ring-hydroxylating naphthalene 1,2-dioxygenase (NahA) enzyme, whose implication in a wide range of different PAHs degradative reactions have been uncovered [69,70], including hydroxylation of anthracene, phenanthrene, and fluorene, and monooxygenation of acenaphthene among others [22,69,71]. Initial oxidation of naphthalene and other PAHs is followed by subsequent reactions until funneled into central aromatic degradation pathways, usually with catechol, gentisate, or protocatechuate as intermediaries depending on the specific PAH (for a review see [20]). The screening of the diesel-degrading consortium metagenome revealed the presence of 83 putative ring-hydroxylating dioxygenases, nine of which were annotated as naphthalene 1,2-dioxygenases (Figure 3a). Most of these NahA were assigned to *Sphingomonas* (5), *Sphingobium* (2), and *Bordetella* (2) (Figure 3b, Supplementary Table S3), which is in agreement with the fraction of *Alphaproteobacteria* present in the microbiome of the consortium growing with phenanthrene as the sole carbon and energy source (Figure 1). Unexpectedly, none of these NahA were assigned to *Pseudomonas* or even to *Gammaproteobacteria*, class that dominates both PAH-degrading microbiomes (59.59% and 99.75% in phenanthrene or naphthalene, respectively). The number of other ring-hydroxylating and ring-cleavage intra and estradiol dioxygenases is also scarce among *Gammaproteobacteria*, which suggest that the involvement of *Pseudomonas* in the degradation of PAHs in the diesel-degrading consortium might be attributed to the use of products of PAHs degradation rather than being involved on its initial oxidation. However, other causes cannot be ruled out, including low representation of enzymes not appearing at the metagenome depth this study was carried out.

Regarding central metabolism of aromatic compounds, catechol 1,2-dioxygenase, catechol 2,3-dioxygenase, gentisate 1,2-dioxygenase, and homogentisate 1,2-dioxygenase were among the most abundant CDSs found in the diesel-degrading metagenome (16, 17, 19 and 19 CDSs respectively, Figure 3a). Protocatechuate 3,4-dioxygenase, protocatechuate 4,5-dioxygenases, and benzoate 1,2-dioxygenase were also found in the metagenome (7, 8, and 10 CDSs, respectively). These results agree with the degradation pathways of the catabolic products of naphthalene, anthracene, phenanthrene, and fluorene, among others, via ortho or meta cleavage [20]. According to the taxonomic assignation of these central aromatic degradation enzymes, most of them belong to genera such as *Pseudomonas*, *Bordetella*, *Achromobacter*, *Sphingomonas*, *Sphingobium*, and *Cupriavidus*, among others (Figure 3b), which could explain their presence in the diesel-degrading consortium.

### 3.6. Metabolic Roles of Specific Populations in the Diesel-Degrading Consortium

**Figure 4:**
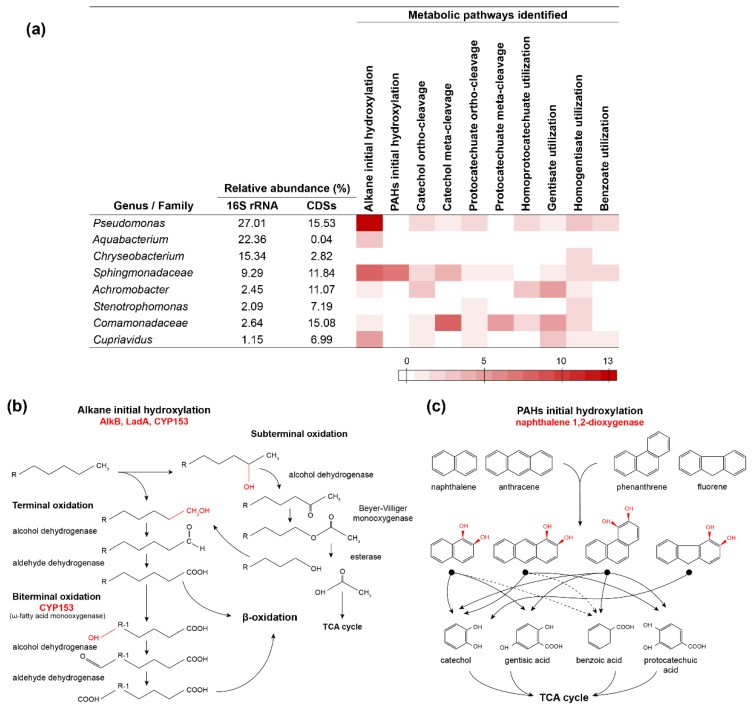
(**a**) Summary of alkanes, PAHs, and central aromatic biodegradation pathways found in the most abundant genera/families within the metagenome of the diesel-degrading consortium. Red scale bar represents the number of enzymes for each pathway identified. (**b**) Schematic view of the initial terminal, subterminal, and biterminal alkane aerobic oxidation mediated by AlkB, LadA, and CYP153 enzymes. Adapted from [7,8]. (**c**) Schematic view of initial naphthalene 1,2-dioxygenase hydroxylating reactions in PAHs components of diesel and aromatic central metabolites generated by further oxidation reactions of these *cis*-diol intermediates. Dotted lines indicate metabolic products depending on specific degradation pathways. Enzymes and chemical reactions catalyzed by these enzymes are indicated in red typing.

The identification and taxonomic assignment of enzymes involved in the initial hydroxylation of alkanes and PAHs within the diesel-degrading consortium metagenome, along with the characterization of the enzymes responsible of central aromatic degradation pathways, provides a profound understanding of the different roles of the main bacterial populations that thrive in the consortium with regard of their relative abundance. These results are summarized in Figure 4. The initial hydroxylation of alkanes is carried out by different bacteria, including *Pseudomonas*, *Aquabacterium*, *Sphingomonadaceae* family bacteria, and *Achromobacter*. Among these, alkane group hydroxylases (AlkB, LadA, and CYP153 cytochrome P450 family) are more abundant in *Pseudomonas* and *Sphingomonadaceae* family, containing 13 and 8 of these enzymes, respectively (Figure 4), which is consistent with their relative abundance in the consortium and with previous reports [34,57,58,59]. These genera could be responsible for the initial terminal or subterminal oxidation or biterminal oxidation of different chain-length alkanes. Conversely, the initial hydroxylation of PAHs based on the presence of naphthalene 1,2-dioxygenases found in the metagenome of the diesel-degrading consortium is primarily attributed to bacteria of the *Sphingomonadaceae* family. High redundancy of central aromatic degradation pathways is observed among the different genera present in the consortium, which could explain the diversity found in the diesel-degrading consortium and the population shift towards *Pseudomonas* when growing in alkanes as sole carbon and energy source (Figure 1). Although none of the naphthalene 1,2-dioxygenases found in the metagenome have been classified as belonging to *Pseudomonas*, the fact that this genus dominates the naphthalene-growing population (Figure 3b), might be also related with the functional redundancy of central aromatic pathways this genus exhibits (Figure 4). Nonetheless, genes from key species playing an important role in the early oxidation of alkanes and PAHs might be missing, since 48 h growth culture of the consortium culture was used to perform the analyses. The different growth pattern of the consortium on different carbon substrates (Supplementary Figure S1) shows that different populations could evolve on time. Further analyses to see the evolution of the community over time and the genes present in different growth stages could provide deeper insights into the biodegradation process.

Interestingly, two of the most abundant genera within the diesel-degrading consortium do not harbor many of the CDSs for these pathways. This is the case of *Aquabacterium* and *Chryseobacterium* (22.36% and 15.34% 16S rRNA relative abundance, respectively). The differences in relative abundance observed between 16S rRNA and CDSs in these genera might suggest a misclassification of CDSs. In the specific case of *Burkholderiales* order, the CDSs abundance of *Commamonadaceae* family and *Cupriavidus* genus (15.08% and 6.99%, respectively) is similar to *Aquabacterium* 16S rRNA (22.36%), another *Burkholderiales* order genus. This finding could explain the relatively low representation of *Aquabacterium* CDSs and its presence in the consortium. Nevertheless, this is not the case of *Chryseobacterium*, a *Flavovacteriia* class whose representation in CDSs is less than 3%. It is unclear if unclassified coding sequences could belong to this genus or the role it might have in the diesel-degrading consortium, even though *Chryseobacterium* have been previously identified in diesel fuel degrading consortia [66]. Other possibilities, such as the bacterial shift towards more metabolically versatile members in late states of the biodegradation process, which do not participate in the initial oxidation of diesel constituents, could also explain the presence of these genera.

### 3.7. Rhizoremediation Assays in Diesel-Polluted Soil Microcosms

**Figure 5:**
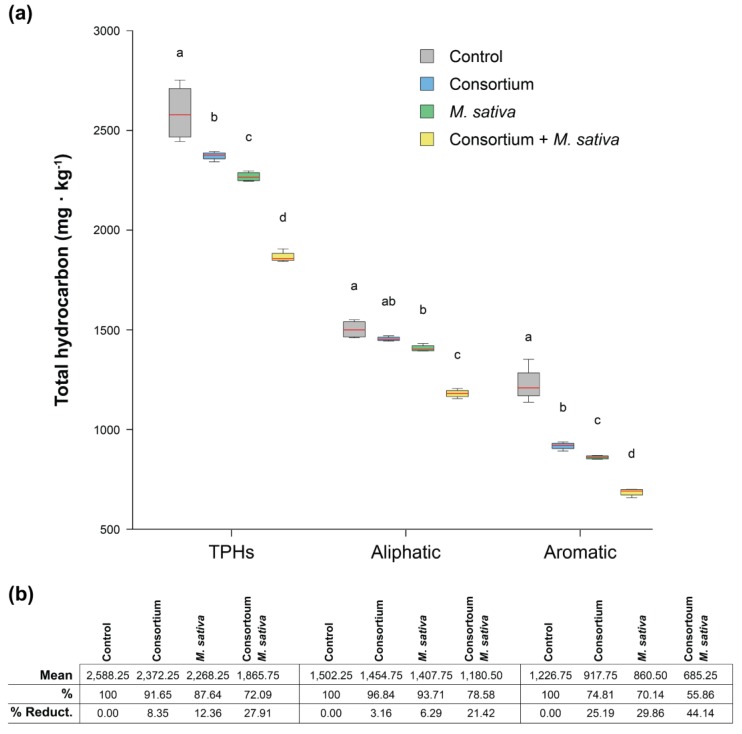
(**a**) Gas chromatography with a flame ionized detector (GC-FID) results of total hydrocarbons remaining after four-month treatments with the diesel-degrading consortium, alfalfa (*M. sativa*) plants without inoculum, and combined consortium and alfalfa plants in four-month microcosms assays. Each box indicates Q1 and Q3, while the red line indicates the median values out of four replicates. Error bars indicate maximum and minimum values. Different letters indicate statistically significant differences (*p* < 0.05) using two-way ANOVA with Tukey’s multiple comparison test corrections. (**b**) Mean values in each treatment in mg·kg^−1^ out of four replicates and percentages compared to the control.

Rhizoremediation with indigenous hydrocarbon-degrading microorganisms have been proposed as one of the most effective techniques in restoring diesel-polluted soils [35,36,38], which could be enhanced by stimulation of the catalytic activities of microorganisms by plant roots [37,72] and can also be combined with other techniques such as chemical oxidation [73]. In order to test whether the bacterial consortium isolated in this study could be suited for rhizoremediation of the original diesel-polluted soil from where it was isolated, soil microcosms assays inoculated with the consortium were evaluated. Additionally, alfalfa (*Medicago sativa*) plants were used to address a possible stimulating effect. The results show a clear soil TPHs reduction after 4 months (Figure 5). In the control, untreated pot, TPHs reduced from the original 2974 mg·kg^−1^ to 2588 mg·kg^−1^. In the soil treatment with the consortium resulted in a further reduction of 8.35%. This reduction was 12.36% with alfalfa plants without inoculum, probably due to the stimulation of indigenous populations already present in the soil. However, the combined effect of the consortium with alfalfa plants resulted in a 27.91% decrement in TPHs, when compared with the original soil, which tripled that of the consortium alone. The aromatic fraction in all cases was the most degraded, showing a 44.14% reduction in the consortium with plants treatment while the aliphatic fraction accounted for a 21.42% reduction (Figure 5). 

Regarding the specific aliphatic and aromatic chain content, the consortium combined with alfalfa plants showed a major reduction of short and long-chain alkanes (>C_10_–C_12_, >C_21_–C_35_ and >C_35_), showing a 65.96%, 27.31%, and 31.36% hydrocarbon reduction respectively (Supplementary Figure S2). These results are consistent with the number of long-chain alkane monooxygenases found in the metagenome and the fact that the consortium is able to grow in the presence of the short-chain alkane hexane. In the case of aromatic hydrocarbons, the diesel-degrading consortium combined with alfalfa plants resulted in a major decrease in most cases (Supplementary Figure S2). This reduction is more substantial in the case of the >EC_10_–EC_16_ fraction (41.67% and 57.69% for >EC_10_–C_12_ and >EC_12_–C_16_, respectively) which is compatible with naphthalene (C_10_), fluorene (C_13_), anthracene (C_14_), and phenanthrene (C_14_), among others. The observed effect caused by alfalfa plants in both, aliphatic and aromatic hydrocarbons was not unexpected, as it has been previously reported [36,38,74] and might be attributed to induction of microbial biodegradation pathways by plant metabolites [75] and other stimulating effects [37,76].

The microcosms results show that the bacterial consortium isolated in this study could serve as an inoculum for effective rhizoremediation of diesel-polluted soils. However, further analyses are required to evaluate its potential in other polluted sites, whose hydrocarbon composition might vary, and to evaluate the plant factors affecting the rhizoremediation process. Additional analyses at different times of the bioremediation process could also provide powerful insights into the community evolution and to identify key bacterial roles of the consortium.

## 4. Conclusions

Complex pollutants such as diesel require multiple microorganisms for their degradation. We have shown here that an effective autochthonous bacterial consortium can be constructed by successive enrichment cultivation of soils from contaminated sites. By metagenomic analysis of the consortium, growing on diesel and on specific aliphatic and polyaromatic hydrocarbons, we have been able to determine the bacterial genera composition of the consortium, the genes and enzymes implicated in diesel degradation and the specific degradative roles of the major populations within the consortium. The functional redundancy observed in the metagenome might be related to the plasticity that allows the populations to adapt to changes in the environment, and therefore conferring robustness to the degrading hydrocarbon system.

## Figures and Tables

**Table 1:** Aliphatic and aromatic hydrocarbon fraction composition of the diesel oil and aged diesel-polluted soil used in this study.

TPH Fraction	Diesel Oil (mg·mL^−1^)	Soil (µg·g^−1^)
Aliphatic hydrocarbons		
>C_10_–C_12_	82 ± 1	3.5 ± 0.5
>C_12_–C_16_	257 ± 7	151 ± 4
>C_16_–C_21_	283 ± 8	563 ± 28
>C_21_–C_35_	55 ± 4	1086 ± 73
>C_35_	0.05 ± 0.001	116 ± 16
Aromatic hydrocarbons		
>EC_10_–C_12_	17 ± 1	11 ± 5
>EC_12_–C_16_	13 ± 1	8 ± 1
>EC_16_–C_21_	57 ± 3	484 ± 48
>EC_21_–C_35_	2 ± 0.1	530 ± 70
>EC_35_	0.1 ± 0.004	22 ± 4
TPHs	764 ± 7	2974 ± 143

## Supplementary Materials

The following are available online at https://www.mdpi.com/2073-4425/10/6/456/s1. Table S1: Relative abundance of the diesel-degrading consortium based on 16S rRNA gene and CDSs from the metagenome annotations. Table S2: Statistics of the 16S rRNA and whole-metagenome shotgun sequencing and processing of reads. Table S3: CDSs for alkanes and PAHs initial hydroxylation and others involved in central aromatic metabolism. Figure S1: Growth curves of the diesel-degrading consortium in each of the substrates used in this study. Figure S2: Detailed GC-FID results of aliphatic and aromatic hydrocarbons fractions remaining after microcosms treatments.
