# Supplementary material for: Metagenomic Insights into the Bacterial Functions of a Diesel-Degrading Consortium for the Rhizoremediation of Diesel-Polluted Soil
Source: Genes (Basel). 2019 Jun 14;10(6):456. doi: 10.3390/genes10060456 (PMC6627497; doi:10.3390/genes10060456)

Supplementary Figure S1. Growth curves of the diesel-degrading consortium in each of the substrates used in this study. Growth was measured by DO600 nm in 10 mL of MM+PAS supplemented with 0.005 (w/v) of yeast extract and 1g/L of each substrate. The lines represent mean values over three replicates.

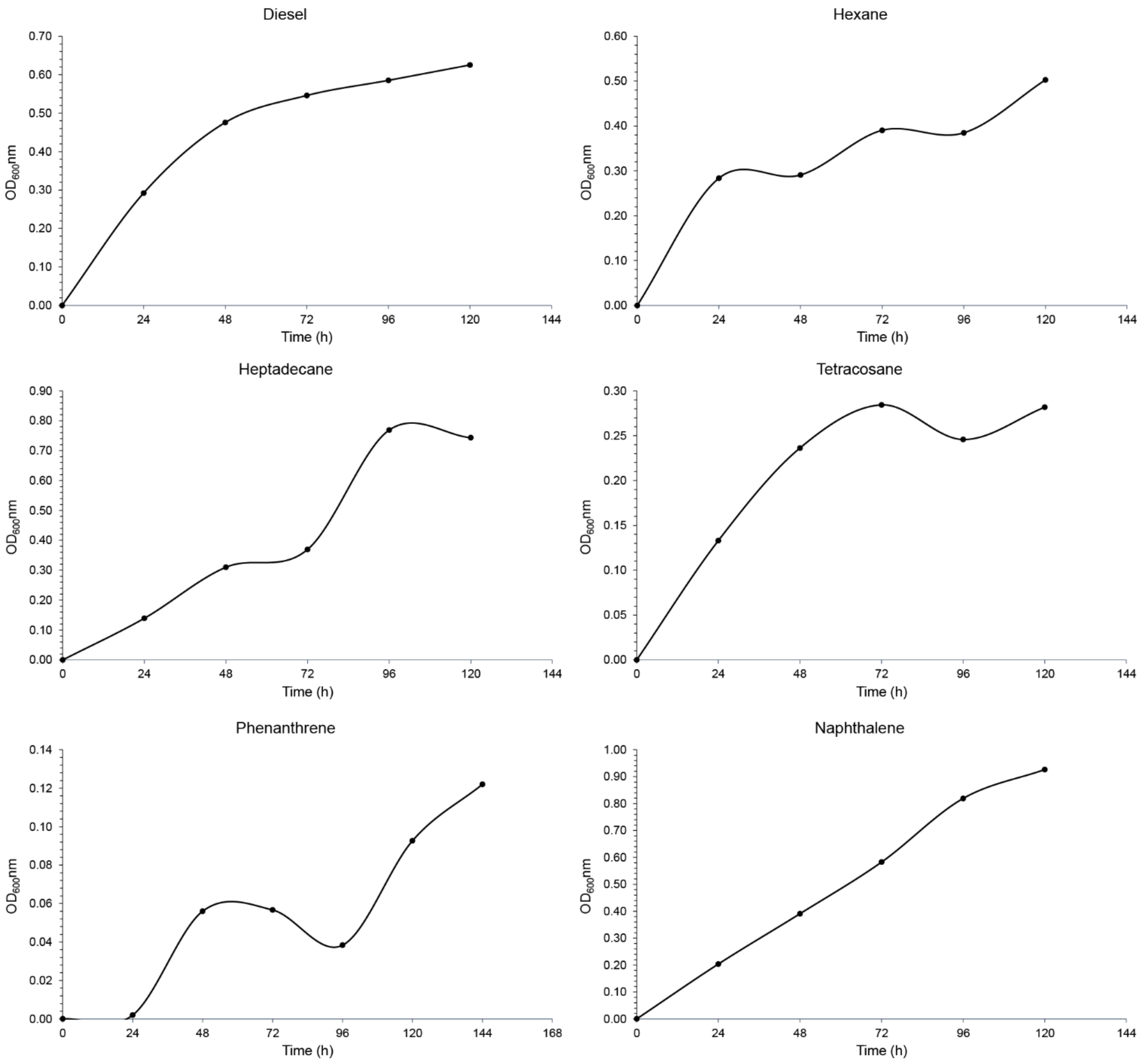

Supplement: Supplementary file 1 [file genes-10-00456-s001.zip › Supplementary_Figure_S1.pdf]
