# Supplementary material for: Metagenomic Insights into the Bacterial Functions of a Diesel-Degrading Consortium for the Rhizoremediation of Diesel-Polluted Soil
Source: Genes (Basel). 2019 Jun 14;10(6):456. doi: 10.3390/genes10060456 (PMC6627497; doi:10.3390/genes10060456)
Supplement: Supplementary file 1 [file genes-10-00456-s001.zip › Supplementary_Figure_S2.pdf]

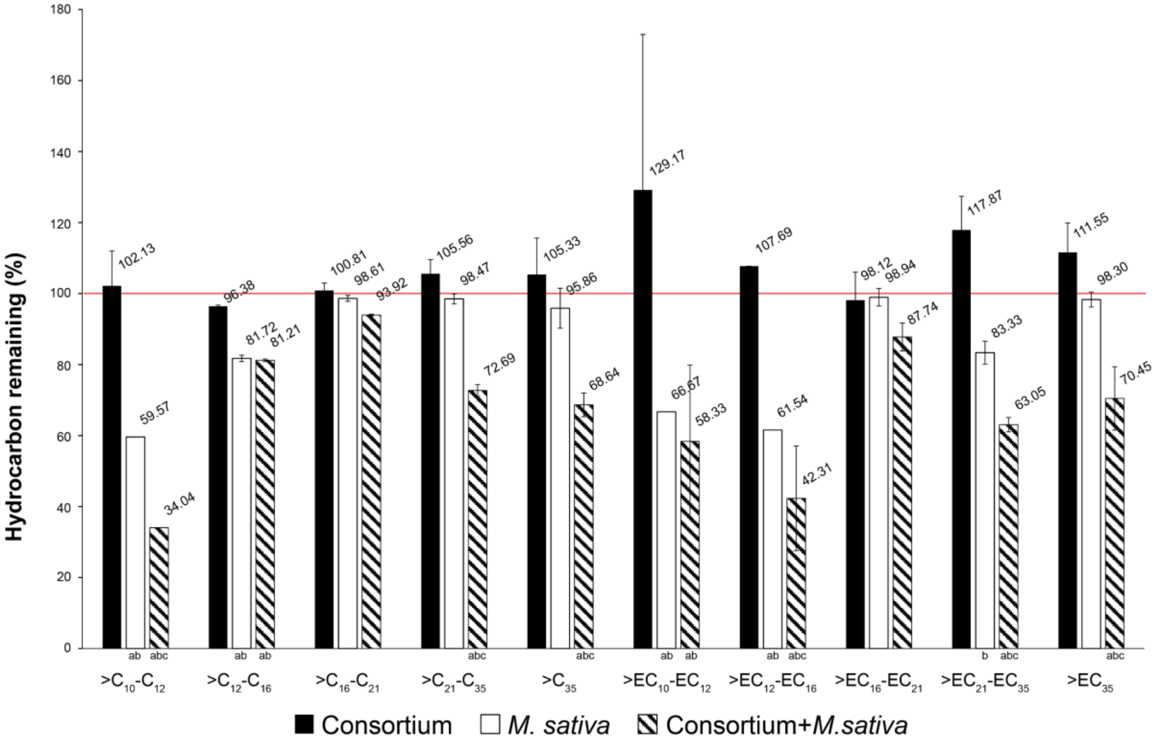

Supplementary Figure S2. GC-FID results of total hydrocarbons fractions remaining after treatments with the diesel-degrading consortium (black), alfalfa (*M. sativa*) plants without inoculum (white) and combined consortium and alfalfa plants (striped). Mean values are detailed at the top of each bar. Error bars indicate standard deviation. The mean of the controls was used as 100%. Different letters at the bottom of each bar indicates statistically significant differences ($p < 0.05$, a; compared with the control, b; compared with the consortium, c; compared with the alfalfa treatment) using two-way ANOVA with Tukey's multiple comparison test correction.
