# Supplementary material for: Metagenomic Insights into the Bacterial Functions of a Diesel-Degrading Consortium for the Rhizoremediation of Diesel-Polluted Soil
Source: Genes (Basel). 2019 Jun 14;10(6):456. doi: 10.3390/genes10060456 (PMC6627497; doi:10.3390/genes10060456)
Supplement: Supplementary file 1 [file genes-10-00456-s001.zip › Supplementary_Table_S1.pdf]

Supplementary Table S1.

Relative abundance of the 16S rRNA sequences at different taxonomic levels retrieved from the diversity analysis of the 16S rRNA.

Taxonomic classification	Relative abundance (%)					
	Diesel	Hexane	Heptadecane	Tetracosane	Phenanthrene	Naphthalene
Bacteria	**100.000**	**100.000**	**100.000**	**100.000**	**100.000**	**100.000**
Acetothermia	**0.000**	**0.000**	**0.000**	**0.000**	**0.000**	**0.005**
Acetothermiia	0.000	0.000	0.000	0.000	0.000	0.005
uncultured bacterium	**0.000**	**0.000**	**0.000**	**0.000**	**0.000**	**0.005**
Acidobacteria	**0.149**	**0.000**	**0.000**	**0.000**	**0.000**	**0.005**
Acidobacteriia	0.149	0.000	0.000	0.000	0.000	0.000
Solibacterales	**0.149**	**0.000**	**0.000**	**0.000**	**0.000**	**0.000**
Solibacteraceae (Subgroup 3)	0.149	0.000	0.000	0.000	0.000	0.000
Bryobacter	0.121	0.000	0.000	0.000	0.000	0.000
Paludibaculum	0.029	0.000	0.000	0.000	0.000	0.000
Thermoanaerobaculia	0.000	0.000	0.000	0.000	0.000	0.005
Thermoanaerobaculales	**0.000**	**0.000**	**0.000**	**0.000**	**0.000**	**0.005**
Thermoanaerobaculaceae	0.000	0.000	0.000	0.000	0.000	0.005
Thermoanaerobaculum	0.000	0.000	0.000	0.000	0.000	0.005
Actinobacteria	**0.000**	**8.695**	**0.004**	**0.000**	**0.004**	**0.000**
Actinobacteria	0.000	8.695	0.004	0.000	0.004	0.000
Corynebacteriales	**0.000**	**8.695**	**0.004**	**0.000**	**0.004**	**0.000**
Nocardiaceae	0.000	8.695	0.004	0.000	0.004	0.000
Gordonia	0.000	8.695	0.004	0.000	0.004	0.000
Bacteroidetes	**16.578**	**0.064**	**1.631**	**4.221**	**0.000**	**0.005**
Bacteroidia	16.578	0.064	1.631	4.221	0.000	0.005
Bacteroidales	**0.000**	**0.000**	**0.000**	**0.000**	**0.000**	**0.005**
Rikenellaceae	0.000	0.000	0.000	0.000	0.000	0.005
Blvii28 wastewater-sludge group	0.000	0.000	0.000	0.000	0.000	0.005
Chitinophagales	**0.185**	**0.000**	**0.048**	**0.316**	**0.000**	**0.000**
Chitinophagaceae	0.185	0.000	0.048	0.316	0.000	0.000
Filimonas	0.084	0.000	0.000	0.313	0.000	0.000
Pseudoflavitalea	0.070	0.000	0.048	0.003	0.000	0.000
Terrimonas	0.031	0.000	0.000	0.000	0.000	0.000
Cytophagales	**0.864**	**0.000**	**0.000**	**0.000**	**0.000**	**0.000**
Spirosomaceae	0.864	0.000	0.000	0.000	0.000	0.000
Dyadobacter	0.864	0.000	0.000	0.000	0.000	0.000
Flavobacteriales	**15.338**	**0.064**	**0.226**	**3.563**	**0.000**	**0.000**
Weeksellaceae	15.338	0.064	0.226	3.563	0.000	0.000
Chryseobacterium	15.338	0.064	0.226	3.563	0.000	0.000
Sphingobacteriales	**0.191**	**0.000**	**1.357**	**0.343**	**0.000**	**0.000**
Sphingobacteriaceae	0.191	0.000	1.357	0.343	0.000	0.000

Nubsella	0.191	0.000	0.010	0.343	0.000	0.000
Sphingobacterium	0.000	0.000	1.347	0.000	0.000	0.000
Cyanobacteria	**0.000**	**0.044**	**0.116**	**0.089**	**0.093**	**0.108**
Oxyphotobacteria	0.000	0.044	0.116	0.089	0.093	0.108
Chloroplast	**0.000**	**0.044**	**0.116**	**0.089**	**0.093**	**0.108**
Epsilonbacteraeota	**0.000**	**0.000**	**0.000**	**0.000**	**0.000**	**0.052**
Campylobacteria	0.000	0.000	0.000	0.000	0.000	0.052
Campylobacterales	**0.000**	**0.000**	**0.000**	**0.000**	**0.000**	**0.052**
Thiovulaceae	0.000	0.000	0.000	0.000	0.000	0.052
Sulfuricurvum	0.000	0.000	0.000	0.000	0.000	0.052
Nitrospirae	**0.000**	**0.000**	**0.000**	**0.000**	**0.000**	**0.052**
Thermodesulfovibrionia	0.000	0.000	0.000	0.000	0.000	0.052
uncultured	**0.000**	**0.000**	**0.000**	**0.000**	**0.000**	**0.052**
Proteobacteria	**83.273**	**91.197**	**98.245**	**95.690**	**99.903**	**99.769**
Alphaproteobacteria	20.225	1.054	0.624	1.107	40.313	0.016
Azospirillales	**1.161**	**0.000**	**0.007**	**0.039**	**0.092**	**0.009**
Azospirillaceae	1.161	0.000	0.007	0.039	0.092	0.009
Azospirillum	0.152	0.000	0.007	0.006	0.006	0.009
Niveispirillum	1.009	0.000	0.000	0.032	0.086	0.000
Caulobacterales	**1.442**	**0.854**	**0.003**	**0.264**	**0.006**	**0.000**
Caulobacteraceae	1.442	0.854	0.003	0.264	0.006	0.000
Brevundimonas	0.178	0.000	0.000	0.199	0.000	0.000
Caulobacter	0.448	0.000	0.003	0.066	0.006	0.000
Phenylobacterium	0.088	0.854	0.000	0.000	0.000	0.000
unknown	0.728	0.000	0.000	0.000	0.000	0.000
Parvibaculales	**3.243**	**0.000**	**0.000**	**0.000**	**0.008**	**0.000**
Parvibaculaceae	3.243	0.000	0.000	0.000	0.008	0.000
Parvibaculum	3.243	0.000	0.000	0.000	0.008	0.000
Rhizobiales	**5.032**	**0.182**	**0.615**	**0.797**	**3.543**	**0.000**
Beijerinckiaceae	2.363	0.000	0.004	0.076	0.050	0.000
Bosea	0.303	0.000	0.000	0.076	0.050	0.000
Camelimonas	2.060	0.000	0.000	0.000	0.000	0.000
Methylobacterium	0.000	0.000	0.004	0.000	0.000	0.000
Kaistiaceae	0.024	0.000	0.000	0.000	0.000	0.000
Kaistia	0.024	0.000	0.000	0.000	0.000	0.000
Labraceae	0.031	0.000	0.323	0.000	2.458	0.000
Labrys	0.031	0.000	0.323	0.000	2.458	0.000
Rhizobiaceae	2.214	0.167	0.281	0.721	1.024	0.000
Allorhizobium-Neorhizobium-Pararhizobium-Rhizobium	0.468	0.021	0.141	0.392	1.016	0.000
Mesorhizobium	0.013	0.000	0.000	0.000	0.000	0.000
Ochrobactrum	0.470	0.000	0.118	0.218	0.009	0.000
Shinella	1.196	0.147	0.022	0.111	0.000	0.000

unknown	0.066	0.000	0.000	0.000	0.000	0.000
Xanthobacteraceae	0.400	0.015	0.007	0.000	0.010	0.000
Blastochloris	0.000	0.000	0.000	0.000	0.003	0.000
Bradyrhizobium	0.000	0.015	0.000	0.000	0.000	0.000
Rhodoplanes	0.000	0.000	0.003	0.000	0.000	0.000
Rhodopseudomonas	0.040	0.000	0.004	0.000	0.008	0.000
Xanthobacter	0.310	0.000	0.000	0.000	0.000	0.000
unknown	0.051	0.000	0.000	0.000	0.000	0.000
Rickettsiales	**0.000**	**0.005**	**0.000**	**0.000**	**0.006**	**0.007**
Mitochondria	0.000	0.005	0.000	0.000	0.006	0.007
unknown	0.000	0.005	0.000	0.000	0.006	0.007
Sneathiellales	**0.057**	**0.000**	**0.000**	**0.000**	**0.000**	**0.000**
Sneathiellaceae	0.057	0.000	0.000	0.000	0.000	0.000
Taonella	0.057	0.000	0.000	0.000	0.000	0.000
Sphingomonadales	**9.290**	**0.013**	**0.000**	**0.008**	**36.659**	**0.000**
Sphingomonadaceae	9.290	0.013	0.000	0.008	36.659	0.000
Novosphingobium	3.645	0.013	0.000	0.008	33.694	0.000
Sphingobium	5.195	0.000	0.000	0.000	2.965	0.000
Sphingomonas	0.279	0.000	0.000	0.000	0.000	0.000
Sphingopyxis	0.171	0.000	0.000	0.000	0.000	0.000
Gammaproteobacteria	63.047	90.143	97.621	94.582	59.589	99.752
Betaproteobacteriales	**29.395**	**0.003**	**5.342**	**4.301**	**5.305**	**0.007**
Burkholderiaceae	29.388	0.003	5.342	4.301	5.305	0.000
Achromobacter	2.453	0.003	4.427	2.164	4.451	0.000
Acidovorax	0.415	0.000	0.000	0.000	0.000	0.000
Aquabacterium	22.355	0.000	0.000	0.000	0.000	0.000
Bordetella	0.018	0.000	0.000	0.000	0.000	0.000
Comamonas	0.000	0.000	0.000	0.013	0.000	0.000
Cupriavidus	1.150	0.000	0.011	0.166	0.854	0.000
Delftia	0.893	0.000	0.880	1.623	0.000	0.000
Diaphorobacter	0.000	0.000	0.000	0.024	0.000	0.000
Hydrogenophaga	0.079	0.000	0.000	0.000	0.000	0.000
Pigmentiphaga	0.620	0.000	0.024	0.251	0.000	0.000
Pseudorhodoferax	1.249	0.000	0.000	0.000	0.000	0.000
Variovorax	0.079	0.000	0.000	0.059	0.000	0.000
unknown	0.077	0.000	0.000	0.000	0.000	0.000
Gallionellaceae	0.000	0.000	0.000	0.000	0.000	0.004
Gallionella	0.000	0.000	0.000	0.000	0.000	0.004
Rhodocyclaceae	0.007	0.000	0.000	0.000	0.000	0.004
Methyloversatilis	0.007	0.000	0.000	0.000	0.000	0.000
uncultured	0.000	0.000	0.000	0.000	0.000	0.004
Enterobacteriales	**0.011**	**0.000**	**0.055**	**0.000**	**0.000**	**0.000**
Enterobacteriaceae	0.011	0.000	0.055	0.000	0.000	0.000

Citrobacter	0.011	0.000	0.000	0.000	0.000	0.000
Enterobacter	0.000	0.000	0.026	0.000	0.000	0.000
unknown	0.000	0.000	0.029	0.000	0.000	0.000
Halothiobacillales	**0.000**	**0.000**	**0.000**	**0.000**	**0.000**	**0.011**
Halothiobacillaceae	0.000	0.000	0.000	0.000	0.000	0.011
Thiofaba	0.000	0.000	0.000	0.000	0.000	0.011
Legionellales	**1.253**	**0.000**	**0.000**	**0.000**	**0.000**	**0.000**
Legionellaceae	1.253	0.000	0.000	0.000	0.000	0.000
Legionella	1.253	0.000	0.000	0.000	0.000	0.000
Pseudomonadales	**27.014**	**64.915**	**88.854**	**89.249**	**53.601**	**99.720**
Pseudomonadaceae	27.014	64.915	88.854	89.249	53.601	99.720
Pseudomonas	27.014	64.915	88.854	89.249	53.601	99.720
Thiotrichales	**0.000**	**0.000**	**0.000**	**0.000**	**0.000**	**0.014**
Thiotrichaceae	0.000	0.000	0.000	0.000	0.000	0.014
Thiothrix	0.000	0.000	0.000	0.000	0.000	0.014
Xanthomonadales	**5.375**	**25.226**	**3.370**	**1.032**	**0.684**	**0.000**
Rhodanobacteraceae	3.289	0.000	0.000	0.006	0.000	0.000
Dokdonella	3.289	0.000	0.000	0.006	0.000	0.000
Xanthomonadaceae	2.086	25.226	3.370	1.026	0.684	0.000
Stenotrophomonas	2.086	25.226	3.370	1.026	0.684	0.000
Unclasified bacteria	**0.000**	**0.000**	**0.004**	**0.000**	**0.000**	**0.004**

Relative abundance of CDSs at different taxonomic levels.

**Taxonomic classification**	**Diesel**
Bacteria	98.32
Actinobacteria	0.33
Actinobacteria	0.33
Corynebacteriales	0.08
Mycobacteriaceae	0.03
Mycobacterium	0.03
Nocardiaceae	0.05

Nocardia	0.02
Rhodococcus	0.03
Frankiales	0.02
Frankiaceae	0.02
Frankia	0.02
Jiangellales	0.01
Jiangellaceae	0.01
Jiangella	0.01
Micrococcales	0.06
Microbacteriaceae	0.06
Microbacterium	0.06
Micromonosporales	0.03
Micromonosporaceae	0.03
Micromonospora	0.03
Pseudonocardiales	0.04
Pseudonocardiaceae	0.04
Amycolatopsis	0.02
Pseudonocardia	0.02
Streptomycetales	0.09
Streptomycetaceae	0.09
Streptomyces	0.09
Bacteroidetes	4.65
Cytophagia	1.66
Cytophagales	1.66
Cytophagaceae	1.66
Dyadobacter	1.66
Flavobacteriia	2.94
Flavobacteriales	2.94
Flavobacteriaceae	2.94
Chryseobacterium	2.82
Elizabethkingia	0.06
Flavobacteriaceae	0.02
Flavobacterium	0.03
Polaribacter	0.01
Sphingobacteriia	0.04
Sphingobacteriales	0.04
Sphingobacteriaceae	0.04
Pedobacter	0.02
Sphingobacterium	0.02
Deinococcus-Thermus	0.01
Deinococci	0.01
Deinococcales	0.01
Deinococcaceae	0.01
Deinococcus	0.01
Proteobacteria	93.33

Alphaproteobacteria	26.38
Caulobacterales	2.32
Caulobacteraceae	2.32
Brevundimonas	0.28
Caulobacter	1.47
Phenylobacterium	0.58
Rhizobiales	10.80
Aurantimonadaceae	0.08
Aureimonas	0.05
Martelella	0.03
Beijerinckiaceae	0.03
Methylocella	0.03
Bradyrhizobiaceae	2.31
Afipia	0.04
Bosea	1.16
Bradyrhizobium	0.75
Nitrobacter	0.03
Oligotropha	0.04
Rhodopseudomonas	0.28
Variibacter	0.02
Brucellaceae	0.46
Brucella	0.08
Ochrobactrum	0.38
Chelatococcaceae	0.53
Chelatococcus	0.53
Hyphomicrobiaceae	0.20
Blastochloris	0.07
Devosia	0.02
Hyphomicrobium	0.02
Rhodomicrobium	0.01
Rhodoplanes	0.07
Methylobacteriaceae	0.74
Methylobacterium	0.63
Microvirga	0.11
Methylocystaceae	0.02
Methylocystis	0.02
Other	0.04
Other	0.04
Phyllobacteriaceae	0.32
Aminobacter	0.04
Chelativorans	0.02
Hoeflea	0.01
Mesorhizobium	0.25
Rhizobiaceae	3.82
Agrobacterium	0.58

Ensifer	0.10
Neorhizobium	0.07
Rhizobium	0.47
Shinella	1.72
Sinorhizobium	0.89
Rhodobiaceae	1.25
Parvibaculum	1.25
Xanthobacteraceae	1.02
Azorhizobium	0.26
Starkeya	0.15
Xanthobacter	0.61
Rhodobacterales	0.22
Hyphomonadaceae	0.02
Hyphomonas	0.02
Rhodobacteraceae	0.21
Celeribacter	0.02
Defluviimonas	0.03
Pannonibacter	0.03
Paracoccus	0.04
Rhodobacter	0.04
Rhodovulum	0.01
Stappia	0.02
Yangia	0.01
Rhodospirillales	1.00
Acetobacteraceae	0.07
Acidiphilium	0.02
Gluconacetobacter	0.01
Roseomonas	0.03
Rhodospirillaceae	0.93
Azospirillum	0.59
Magnetospirillum	0.07
Rhodospirillum	0.20
Tistrella	0.08
Sphingomonadales	11.96
Erythrobacteraceae	0.12
Altererythrobacter	0.04
Croceicoccus	0.02
Erythrobacter	0.04
Porphyrobacter	0.02
Sphingomonadaceae	11.84
Blastomonas	0.04
Citromicrobium	0.02
Novosphingobium	3.49
Sphingobium	4.12
Sphingomonas	1.28

Sphingopyxis	2.89
unclassified	0.07
unclassified	0.07
Polymorphum	0.07
Betaproteobacteria	39.32
Burkholderiales	38.92
Alcaligenaceae	13.08
Achromobacter	11.07
Bordetella	1.87
Castellaniella	0.11
Pigmentiphaga	0.03
Burkholderiaceae	8.91
Burkholderia	0.85
Cupriavidus	6.99
Pandoraea	0.10
Paraburkholderia	0.04
Ralstonia	0.93
Comamonadaceae	15.08
Acidovorax	2.62
Alicycliphilus	0.62
Comamonas	0.26
Curvibacter	0.01
Delftia	6.19
Hydrogenophaga	0.93
Limnohabitans	0.02
Other	0.02
Ottowia	0.01
Polaromonas	0.19
Ramlibacter	0.85
Rhodoferax	0.97
Variovorax	2.29
Verminephrobacter	0.10
Oxalobacteraceae	0.31
Collimonas	0.03
Herbaspirillum	0.08
Janthinobacterium	0.05
Massilia	0.14
unclassified	1.47
Aquabacterium	0.04
Leptothrix	0.37
Methylibium	0.45
Mitsuaria	0.17
Roseateles	0.13
Rubrivivax	0.32
unclassifiedBurkholderiales	0.06

Paucibacter	0.06
unkclassified	0.02
Thiomonas	0.02
Neisseriales	0.10
Chromobacteriaceae	0.10
Chromobacterium	0.04
Jeongeupia	0.02
Pseudogulbenkiania	0.02
Vogesella	0.02
Nitrosomonadales	0.05
Sterolibacteriaceae	0.05
Methyloversatilis	0.04
Sulfuritalea	0.01
Rhodocyclales	0.23
Azonexaceae	0.02
Dechloromonas	0.02
Rhodocyclaceae	0.02
Dechlorosoma	0.02
Zoogloeaceae	0.19
Azoarcus	0.12
Thauera	0.07
unclassified	0.02
unclassified	0.02
Betaproteobacteria	0.02
Deltaproteobacteria	0.35
Myxococcales	0.35
Anaeromyxobacteraceae	0.01
Anaeromyxobacter	0.01
Myxococcaceae	0.03
Myxococcus	0.03
Polyangiaceae	0.31
Polyangium	0.30
Sorangium	0.01
Gammaproteobacteria	27.28
Aeromonadales	0.01
Aeromonadaceae	0.01
Aeromonas	0.01
Chromatiales	0.01
Ectothiorhodospiraceae	0.01
Thioalkalivibrio	0.01
Enterobacterales	0.04
Enterobacteriaceae	0.03
Klebsiella	0.03
Yersiniaceae	0.02
Serratia	0.02

Immundisolibacterales	0.01
Immundisolibacteraceae	0.01
Immundisolibacter	0.01
Legionellales	0.14
Legionellaceae	0.14
Legionella	0.12
Tatlockia	0.02
Oceanospirillales	0.05
Alcanivoracaceae	0.02
Alcanivorax	0.02
Halomonadaceae	0.03
Halomonas	0.02
Halotalea	0.01
Pseudomonadales	15.64
Pseudomonadaceae	15.64
Azotobacter	0.11
Pseudomonas	15.53
Xanthomonadales	11.36
Rhodanobacteraceae	3.54
Dokdonella	3.45
Dyella	0.06
Rhodanobacter	0.03
Xanthomonadaceae	7.82
Luteimonas	0.02
Lysobacter	0.20
Pseudoxanthomonas	0.14
Stenotrophomonas	7.19
Xanthomonas	0.28
**Other**	1.68
