# Supplementary material for: Metagenomic Insights into the Bacterial Functions of a Diesel-Degrading Consortium for the Rhizoremediation of Diesel-Polluted Soil
Source: Genes (Basel). 2019 Jun 14;10(6):456. doi: 10.3390/genes10060456 (PMC6627497; doi:10.3390/genes10060456)
Supplement: Supplementary file 1 [file genes-10-00456-s001.zip › Supplementary_Table_S2.pdf]

Supplementary Table S2. Statistics of the 16S rRNA microbiome sequencing and processing of reads

	16S rRNA					
	**Diesel**	**Hexane**	**Heptadecane**	**Tetracosane**	**Naphthalene**	**Phenanthrene**
Paired-end raw reads	112,255	193,494	165,502	126,957	84,263	139,585
Trimmed reads	107,885	186,487	161,310	123,670	80,833	135,330
Processed data						
Filtered	46,341	124,290	106,631	80,773	57,154	93,605
Denoised	46,259	124,088	106,581	80,706	57,086	93,524
Merged	45,805	122,342	106,394	78,213	55,737	93,404
Non-chimeric	45,488	115,347	105,747	77,581	55,737	79,263
Total ASVs	76	29	45	45	20	34

Statistics of the whole-metagenome shotgun sequencing and processing of reads

	**Whole-metagenome shotgun**
Paired-end raw reads	4,251,428
Trimmed reads	4,130,449
Processed data	
Total contigs	114,357
Contigs > 1Kb	18,473
Largest contig (pb)	823,847
Total length (bp)	140,012,582
N50	3,237
GC%	64.03
N's	0
CDSs	120,867
Assigned CDSs	78,110
16S rDNA	53
