# Supplementary material for: Metagenomic Insights into the Bacterial Functions of a Diesel-Degrading Consortium for the Rhizoremediation of Diesel-Polluted Soil
Source: Genes (Basel). 2019 Jun 14;10(6):456. doi: 10.3390/genes10060456 (PMC6627497; doi:10.3390/genes10060456)
Supplement: Supplementary file 1 [file genes-10-00456-s001.zip › Supplementary_Table_S3.pdf]

Supplementary Table S3. Enzymes identified, and genus assignation retrieved from the metagenome of the diesel-degrading bacterial consortium

Contig ID	Start	End	Strand	Size (bp)	Relevant closest relative			Genus assignation
					Organism	% identity	% coverage	
**Alkyl group hydroxylases**								
**Alkane-1 monooxygenase (EC 1.14.15.3)**								
6	166087	167259	+	1173	*Pseudomonas mendocina* ymp	84%	93%	*Pseudomonas*
10	173320	174468	+	1149	*Pseudomonas aeruginosa* DK2	100%	100%	*Pseudomonas*
28	200801	201937	-	1137	*Pseudomonas aeruginosa* Pa58	86%	95%	*Pseudomonas*
155	32852	34018	+	1167	*Aquabacterium parvum* B6	64%	99%*	*Aquabacterium*
213	6803	7966	+	1164	*Pseudomonas* sp. HAR-UPW-AIA-41	75%	97%*	*Pseudomonas*
213	8720	9907	-	1188	*Aquabacterium parvum* B6	96%	100%*	*Aquabacterium*
213	12868	13122	-	255	*Aquabacterium parvum* B6	94%	94%*	*Aquabacterium*
257	24756	25889	+	1134	*Pseudomonas aeruginosa* Pa1242	100%	100%	*Pseudomonas*
2908	2582	3685	+	1104	*Sphingobium* sp. PNB	100%	100%	*Shingobium*
6706	194	1090	-	897	*Sphingomonas* sp. P2	100%	100%	*Sphingomonas*
							**Total**	**10**
**Long-chain alkane monooxygenase (EC 1.14.14.5)**								
1	175638	176786	-	1149	*Pseudomonas aeruginosa* C-NN2	100%	100%	*Pseudomonas*
10	141266	142348	+	1083	*Pseudomonas aeruginosa* NHmuc	100%	100%	*Pseudomonas*
10	143312	144394	+	1083	*Pseudomonas aeruginosa* Ocean-1155	100%	100%	*Pseudomonas*
28	61150	62298	-	1149	*Pseudomonas resinovorans* NBRC 106553	92%	98%	*Pseudomonas*
49	124320	125399	+	1080	*Pseudomonas aeruginosa* PA83	99%	100%	*Pseudomonas*
70	59224	60369	+	1146	*Pseudomonas aeruginosa* Pa84	100%	100%	*Pseudomonas*
76	81011	81949	+	939	*Novosphingobium* sp. AP12	82%	100%*	*Novosphingobium*
76	81964	83118	+	1155	*Sphingomonas* sp. MM-1	77%	96%	*Sphingomonas*
142	56674	57831	+	1158	*Pseudomonas resinovorans* NBRC 106553	92%	97%	*Pseudomonas*
189	34214	35338	-	1125	-			Unassigned
336	8193	9386	+	1194	*Rhizobacter gummiphilus* NS21	86%	96%	*Rhizobacter*
429	17394	18482	+	1089	*Sphingobium* sp. YBL2	85%	97%	*Sphingobium*
609	9134	10231	-	1098	*Sphingomonas taxi* ATCC 55669	81%	98%	*Sphingomonas*
616	13791	14888	+	1098	*Cupriavidus nantongensis* X1	93%	100%	*Cupriavidus*
662	117	1109	+	993	*Pseudomonas citronellolis* SJTE-3	83%	97%	*Pseudomonas*
698	4296	5387	-	1092	*Bordetella pseudohinzii* HI4681	81%	97%	*Bordetella*
1242	3909	5108	+	1200	*Mesorhizobium loti* TONO	77%	86%	*Mesorhizobium*
1557	2241	3302	-	1062	*Rhizobium legominosarum* Vaf-108	76%	95%	*Rhizobium*
2589	1108	2271	-	1164	*Cupriavidus* sp. N-1	92%	100%	*Cupriavidus*
8833	16	1425	+	1410	*Caulobacter segnis* ATCC 21756	85%	77%	*Caulobacter*
16274	61	978	+	918	*Xanthobacter autotrophicus* Py2	81%	99%	*Xanthobacter*
27781	487	750	-	264	*Delftia acidovorans* ANG1	99%	100%	*Delftia*
31735	537	686	-	150	*Bordetella hinzii* H568	85%	70%	*Bordetella*
32955	233	652	-	420	*Niveispirillum cyanobacteriorum* TH16	97 %	66%*	*Niveispirillum*
40409	128	595	+	468	*Cupriavidus nantongensis* X1	83%	55%	*Cupriavidus*
40618	41	571	-	531	*Sphingomonas* sp. 66-10	81%	100%*	*Sphingomonas*
80049	11	277	+	267	*Achromobacter xylosoxidans* NCTC10807	98%	100%	*Achromobacter*
85652	103	381	-	279	*Niveispirillum cyanobacteriorum* TH16	91 %	100%*	Niveispirillum
91760	5	253	+	249	*Cupriavidus* sp. USMAA1020	79%	63%	*Cupriavidus*
							**Total**	**29**
**Cytochrome P450 / Alkane hydroxylase (EC 1.14.-.-)**								
9078	387	1643	-	1257	*Sphingobium macrogoltabida* HXN-200	97%	100%	*Sphingobium*
2344	2533	3798	+	1266	*Parvibaculum lamentivorans* DS-1	77%	96%	*Parvibaculum*
323	1307	2560	+	1254	*Parvibaculum lamentivorans* DS-1	94%	100%	*Parvibaculum*
2593	3099	4364	+	1266	*Parvibaculum lamentivorans* DS-1	98%	100%	*Parvibaculum*
1052	2737	4002	+	1266	*Parvibaculum lamentivorans* DS-1	96%	100%	*Parvibaculum*
445	2891	4171	+	1281	*Parvibaculum lamentivorans* DS-1	90%	86%	*Parvibaculum*
1645	1782	3035	-	1254	*Cupriavidus* sp. K31	76%	72%	*Cupriavidus*
84	45064	46602	+	1539	-			Unassigned
							**Total**	**8**
**Methane, Butane and Propane Monooxygenases (EC 1.14.13.25)**								
662	9850	11511	+	1661	*Methylibium petroleiphillum* PM1	92%	99%	*Methylibium*
							**Total**	**1**
**Extradiol aromatic-ring-cleavage dioxygenases**								
**Catechol 2,3-dioxygenase (EC 1.13.11.2)**								
94	3098	4000	-	903	*Sphingomonas sanxanigenens* NX02	84%	99%	*Sphingomonas*
286	28935	29837	+	903	*Sphingomonas sanxanigenens* NX02	84%	100%	*Sphingomonas*
1168	10485	11480	-	996	*Delftia tsuruhatensis* CM13	77%	90%	*Delftia*
1320	3582	4535	-	954	-			Unassigned
1985	6151	7095	-	945	*Variovorax boronicumulans* J1	78%	84%	*Variovorax*
2305	119	1060	-	942	-			Unassigned
2395	5777	6283	+	507	*Variovorax boronicumulans* J1	83%	93%	*Variovorax*
2880	2921	3865	+	945	*Variovorax boronicumulans* J1	78%	84%	*Variovorax*
3266	2093	3016	+	924	*Sphingomonas* sp. DN1	99%	100%	*Sphingomonas*
13885	77	1054	+	978	*Delftia tsuruhatensis* CM13	99%	100%	*Delftia*
39506	421	612	-	192	-			Unassigned
68221	73	201	+	129	*Variovorax boronicumulans* J1	97%	100%	*Variovorax*
4176	1509	2411	+	903	-			Unassigned

4512	1967	2509	+	543	*Variovorax* sp. HW608 ǀ 76% ǀ 76%	*Variovorax*
35292	22	579	+	558	*Variovorax paradoxus* B4	*Variovorax*
57	13059	13472	-	414	*Pseudomonas resinovorans* NBRC 106553 ǀ 84% ǀ 94%	*Pseudomonas*
148	17832	18767	+	936	*Sphingobium* sp. EP60837 ǀ 81% ǀ 94%	*Sphingobium*
					**Total**	**17**
	**Biphenyl-2,3-diol 1,2-dioxygenase (EC 1.13.11.39)**					
31	61506	62048	-	543	-	Unassigned
547	9686	10630	+	945	-	Unassigned
820	9954	10424	-	471	*Achromobacter xylosoxidans* A8 ǀ 80% ǀ 94%	*Achromobacter*
877	4763	5326	-	564	-	Unassigned
1144	9750	10322	-	573	-	Unassigned
2260	2755	3234	-	480	*Acidovorax avenae* ATCC 19869 ǀ 82% ǀ 95%	*Acidovorax*
2322	146	616	-	471	-	Unassigned
32214	86	664	+	579	*Sphingopyxis* sp. UC10 ǀ 95% ǀ 100%	*Sphingopyxis*
32	165870	166250	-	381	*Pseudomonas aeruginosa* NHmuc ǀ 100% ǀ 100%	*Pseudomonas*
1650	2193	2573	+	381	*Achromobacter denitrificans* PR1 ǀ 99% ǀ 100%	*Achromobacter*
2007	3764	4144	-	381	*Alicycliphilus denitrificans* K601 ǀ 83% ǀ 96%	*Alicycliphilus*
31	70052	71191	+	1140	-	Unassigned
1124	4188	5087	-	900	*Sphingobium* sp. PNB ǀ 99% ǀ 100%	*Sphingobium*
1283	7482	8438	+	957	*Burkholderia cepacia* R34 ǀ 76% ǀ 98%	*Burkholderia*
1824	4253	5152	+	900	*Sphingomonas* sp. DN1 ǀ 100% ǀ 100%	*Sphingomonas*
2335	943	1527	-	585	*Cupriavidus basilensis* 4G11 ǀ 82% ǀ 90%	*Cupriavidus*
3841	1379	2311	-	933	*Bordetella* sp. SCN 67-23 ǀ 94% ǀ 98%*	*Bordetella*
46718	168	563	-	396	*Parvibaculum lavamentivorans* DS-1 ǀ 100% ǀ 100%	*Parvibaculum*
68805	73	399	+	327	-	Unassigned
85055	109	339	-	231	*Phenylobacterium* sp. Root1277 ǀ 91% ǀ 100%	*Phenylobacterium*
36	199468	200358	+	891	*Pseudomonas resinovorans* NBRC 106553 ǀ 89% ǀ 100%	*Pseudomonas*
532	10366	11280	+	915	*Sphingobium* sp. EP60837 ǀ 81% ǀ 98%	*Sphingobium*
746	5908	6789	+	882	-	Unassigned
1382	8342	9307	+	966	*Bordetella* sp. SCN 67-23 ǀ 94% ǀ 100%*	*Bordetella*
1730	5146	5973	+	828	*Bordetella* sp. SCN 67-23 ǀ 87% ǀ 100%*	*Bordetella*
16922	14	1048	-	1035	*Acidovorax* sp. KKS102 ǀ 98% ǀ 100%	*Acidovorax*
22706	19	429	+	411	*Achromobacter denitrificans* A41 ǀ 87% ǀ 100%	*Achromobacter*
					**Total**	**27**
	**3-carboxyethylcatechol 2,3-dioxygenase (EC 1.13.11.16)**					
544	3324	4283	+	960	*Bordetella* sp. SCN 67-23 ǀ 94% ǀ 99%*	*Bordetella*
621	12338	13423	+	1086	-	Unassigned
715	10683	11630	+	948	-	Unassigned
1036	9719	10693	-	975	*Achromobacter denitrificans* PR1 ǀ 99% ǀ 100%	*Achromobacter*
1946	6387	7481	+	1095	*Bordetella* sp. SCN 67-23 ǀ 98% ǀ 100%*	*Bordetella*
1984	7205	7546	-	342	*Bordetella* sp. SCN 67-23 ǀ 96% ǀ 100%*	*Bordetella*
6446	1021	2094	-	1074	*Bordetella* sp. SCN 67-23ǀ 96% ǀ 100%	*Bordetella*
					**Total**	**7**
	**3-hydroxyantrhanilate 3,4-dioxygenase (EC 1.13.11.6)**					
25	29738	30268	+	531	*Dockdonella koreensis* DS-123 ǀ 91% ǀ 97%	*Dockdonella*
141	2980	3552	+	573	*Pseudomonas* sp. St29 ǀ 89% ǀ 97%	*Pseudomonas*
1169	5910	6461	+	552	*Achromobacter denitrificans* PR1 ǀ 97% ǀ 96%	*Achromobacter*
4136	1703	2212	+	510	*Bordetella* sp. SCN 67-23*	*Bordetella*
4675	2913	3260	-	348	*Pseudorhodoferax* sp. Leaf265 ǀ 95% ǀ 100%*	*Pseudorhodoferax*
4751	453	986	+	534	*Bordetella* sp. SCN 67-23*	*Bordetella*
7407	244	765	+	522	*Stenotrophomonas maltophilia* AA1 ǀ 96% ǀ 100%	*Stenotrophomonas*
21428	99	620	+	522	*Sphingopyxis macrogoltabida* EY-1 ǀ 84% ǀ 99%	*Sphingopyxis*
28435	138	467	+	330	*Sphingopyxis macrogoltabida* 203N ǀ 97% ǀ 100%	*Sphingopyxis*
61133	11	241	+	231	*Achromobacter xylosoxidans* NCTC10807 ǀ 99% ǀ 100%	*Achromobacter*
					**Total**	**10**
	**3-O-methylgallate 3,4-dioxygenase (EC 1.13.11.-)**					
1545	8101	9036	-	936	*Bordetella* sp. SCN 67-23 ǀ 95% ǀ 100%*	*Bordetella*
108283	45	317	+	273	*Novosphingobium* sp. Leaf2 ǀ 76% ǀ 100%	*Novosphingobium*
					**Total**	**2**
	**2,3-dihydryphenylpropionate 1,2-dioxygenase (EC 1.13.11.-)**					
106971	54	356	-	303	*Pseudomonas putida* GM4FR ǀ 87% ǀ 100%	*Pseudomonas*
					**Total**	**1**
	**4,5-DOPA dioxygenase (EC 1.14.99.-)**					
159	42000	42377	+	378	*Pseudomonas aeruginosa* Pa127 ǀ 100% ǀ 100%	*Pseudomonas*
2257	5322	5675	-	354	*Achromobacter denitrificans* PR1 ǀ 99% ǀ 100%	*Achromobacter*
3101	3005	3388	+	384	*Achromobacter xyosoxidans* FDAARGOS ǀ 77% ǀ 84%	*Achromobacter*
69822	28	351	+	324	-	Unassigned
2874	5077	5349	-	273	*Bordetella* sp. SCN 67-23 ǀ 96% ǀ 100%*	*Bordetella*
3	44413	44781	+	369	*Pseudomonas resinovorans* NBRC 106553 ǀ 86% ǀ 93%	*Pseudomonas*
					**Total**	**6**
	**2-aminophenol-1,6-dioxygenase (EC 1.13.11.n1)**					
340	24548	25366	+	819	*Achromobacter denitrificans* PR1 ǀ 99% ǀ 100%	*Achromobacter*
7580	1107	1919	-	813	*Bordetella* sp. SCN 67-23 ǀ 96% ǀ 100%*	*Bordetella*
27574	1	411	+	411	*Achromobacter xylosoxidans* A8 ǀ 93% ǀ 100%	*Achromobacter*
					**Total**	**3**
	**Protocatechuate 4,5-dioxygenase (EC 1.13.11.8)**					
244	25742	26197	-	456	*Sphingobium* sp. C1 ǀ 87% ǀ 98%	*Sphingobium*
1334	63	521	-	459	*Delftia* sp. Cs1-4 ǀ 99% ǀ 100%	*Delftia*
2020	1809	2174	+	366	-	Unassigned

3015	493	855	-	363	-	Unassigned
3471	697	1143	-	447	*Variovorax* sp. HW608 ǀ 87% ǀ 89%	*Variovorax*
5183	18	281	+	264	*Delftia acidovorax* ANG1 ǀ 99% ǀ 100%	*Delftia*
12427	746	1186	-	441	*Delftia tsuruhatensis* CM13 ǀ 89% ǀ 96%	*Delftia*
79107	61	375	-	315	*Delftia tsuruhatensis* CM13 ǀ 99% ǀ 100%	*Delftia*
					**Total**	8
	**3,4-dihydroxyphenylacetate 2,3-dioxygenase (EC 1.13.11.15)**					
170	29369	30379	+	1011	*Achromobacter denitrificans* PR1 ǀ 99% ǀ 100%	*Achromobacter*
6	68470	69393	+	924	*Pseudomonas resinovorans* NBRC 106553 ǀ 90% ǀ 99%	*Pseudomonas*
43	85211	86134	-	924	*Pseudomonas aeruginosa* X78812 ǀ 100% ǀ 100%	*Pseudomonas*
441	7258	8109	+	852	*Achromobacter denitrificans* PR1 ǀ 99% ǀ 100%	*Achromobacter*
1518	6227	7105	-	879	*Acidovorax avenae* ATCC 19860 ǀ 82% ǀ 97%	*Acidovorax*
66678	158	436	-	279	*Comamonas* sp. SCN 65-56 ǀ 83% ǀ 100%*	*Comamonas*
70311	21	239	+	219	*Achromobacter xylosoxidans* FDAARGOS ǀ 99% ǀ 100%	*Achromobacter*
					**Total**	**7**
	**Intradiol aromatic-ring-cleavage dioxygenases**					
	**Catechol 1,2-dioxygenase (EC 1.13.11.1)**					
10	254071	255003	+	933	*Pseudomonas aeruginosa* NHmuc ǀ 100% ǀ 100%	*Pseudomonas*
89	65651	66580	-	930	*Pseudomonas pseudoalcaligenes* KF707 ǀ 87% ǀ 94%	*Pseudomonas*
313	28152	29072	+	921	*Sphingobium* sp. TKS ǀ 87% ǀ 99%	*Sphingobium*
503	1138	2058	-	921	*Novosphingobium resinovorum* SA1 ǀ 88% ǀ 95%	*Novosphingobium*
2619	1214	2083	+	870	*Bordetella* sp. SCN 67-23 ǀ 98% ǀ 100%*	*Bordetella*
2693	3014	3889	+	876	*Burkholderia cenocepacia* VC1254 ǀ 77% ǀ 97%	*Burkholderia*
3582	3094	4014	+	921	*Cupriavidus nantongensis* X1 ǀ 95% ǀ 100%	*Cupriavidus*
5050	743	1693	+	951	*Bordetella* sp. SCN 67-23 ǀ 95% ǀ 99%*	*Bordetella*
54674	264	398	-	135	*Mesorhizobium loti* ǀ 76% ǀ 95%*	*Mesorhizobium*
70999	86	415	-	330	*Bosea thiooxidans* CGMCC 9174 ǀ 100% ǀ 100%*	*Bosea*
95825	179	304	-	126	*Mesorhizobium ciceri* WSM1271 ǀ 78% ǀ 100%*	*Mesorhizobium*
111855	145	300	-	156	*Achromobacter insolitus* DSM 23807 ǀ 96% ǀ 100%	*Achromobacter*
447	23817	24689	+	873	*Achromobacter denitrificans* PR1 ǀ 99% ǀ 100%	*Achromobacter*
32983	56	634	+	579	*Variovorax paradoxus* EPS ǀ 92% ǀ 96%	*Variovorax*
37018	38	481	+	444	*Achromobacter xylosoxidans* A8 ǀ 92% ǀ 99%	*Achromobacter*
40315	6	512	+	507	-	Unassigned
					**Total**	**16**
	**Protocatechuate 3,4-dioxygenase (EC 1.13.11.3)**					
87	49809	50414	-	606	*Pseudomonas aeruginosa* M28A1 ǀ 100% ǀ 100%	*Pseudomonas*
470	9873	10478	-	606	*Pseudomonas pseudoalcaligenes* KF707 ǀ 88% ǀ 100%	*Pseudomonas*
1861	2746	3315	+	570	*Cupriavidus nantongensis* X1 ǀ 93% ǀ 100%	*Cupriavidus*
4543	1592	2176	-	585	*Ralstonia solanacearum* CFBP2957 ǀ 81% ǀ 85%	*Ralstonia*
8475	363	926	-	564	*Stenotrophomonas maltophilia* AA1 ǀ 93% ǀ 100%	*Stenotrophomonas*
818	4952	5842	-	891	*Novosphingobium* sp. P6W ǀ 85% ǀ 100%*	*Novosphingobium*
6186	581	1507	+	927	*Delftia* sp. HK171 ǀ 98% ǀ 100%	*Delftia*
					**Total**	**7**
	**Oher aromatic-ring-cleavage dioxygenases**					
	**Gentisate 1,2-dioxygenase (EC 1.13.11.4)**					
10	290582	291643	-	1062	*Pseudomonas aeruginosa* DK2 ǀ 100% ǀ 100%	*Pseudomonas*
240	37602	38678	+	1077	*Achromobacter denitrificans* PR1 ǀ 99% ǀ 100%	*Achromobacter*
269	337	1461	-	1125	*Achromobacter denitrificans* PR1 ǀ 99% ǀ 100%	*Achromobacter*
539	10893	11945	+	1053	*Achromobacter denitrificans* PR1 ǀ 99% ǀ 100%	*Achromobacter*
639	2558	3604	+	1047	-	Unassigned
810	1202	2392	-	1191	*Acidovorax avenae* ATCC 19860 ǀ 83% ǀ 89%	*Acidovorax*
999	3394	4518	+	1125	*Variovorax* sp. HW608 ǀ 81% ǀ 96%	*Variovorax*
1250	27	221	+	195	*Bordetella* sp. SCN 67-23 ǀ 98% ǀ 100%*	*Bordetella*
1687	997	2028	-	1032	*Bordetella* sp. SCN 67-23 ǀ 97% ǀ 100%*	*Bordetella*
1852	31	153	+	123	*Cupriavidus nantongensis* X1 ǀ 98% ǀ 100%	*Cupriavidus*
4783	2758	3216	-	459	*Acidovorax* sp. KKS102 ǀ 97% ǀ 95%	*Acidovorax*
5573	1865	2377	-	513	*Acidovorax wautersii* DSM 27981 ǀ 75% ǀ 98%*	*Acidovorax*
5758	100	1026	+	927	*Achromobacter ruhlandii* SCCH3:ACH ǀ 85% ǀ 100%	*Achromobacter*
14794	11	238	+	228	*Cupriavidus* sp. UYPR2.512 ǀ 81% ǀ 100%*	*Cupriavidus*
16309	16	726	+	711	*Achromobacter xylosoxidans* NH44784-1996 ǀ 96% ǀ 100%	*Achromobacter*
17111	75	1043	-	969	*Delftia tsuruhatensis* CM13 ǀ 98% ǀ 100%	*Delftia*
32039	331	645	-	315	*Cupriavidus* sp. USMAA1020 ǀ 84% ǀ 97%	*Cupriavidus*
32821	37	639	+	603	*Ensifer sojae* CCBAU 05684 ǀ 80% ǀ 95%	*Ensifer*
98422	31	318	-	288	*Sphingopyxis macrogoltabida* 203N ǀ 93% ǀ 100%	*Sphingopyxis*
					**Total**	**19**
	**Homogentisate 1,2-dioxygenase (EC 1.13.11.5)**					
7	13333	14520	+	1188	*Chryseobacterium* sp. StRB126 ǀ 84% ǀ 96%	*Chryseobacterium*
49	25979	27277	-	1299	*Pseudomonas aeruginosa* NHmuc ǀ 100% ǀ 100%	*Pseudomonas*
54	115589	116878	-	1290	*Sphingobium* sp. C1 ǀ 82% ǀ 97%	*Sphingobium*
81	107824	109131	-	1308	*Dokdonella koreensis* DS-123 ǀ 88% ǀ 98%	*Dokdonella*
145	20513	21841	+	1329	*Sphingomonas hengshuiensis* WHSC-8 ǀ 82% ǀ 95%	*Sphingomonas*
254	33500	34792	+	1293	*Pseudomonas citronellolis* P3B5 ǀ 89% ǀ 98%	*Pseudomonas*
614	21	1352	+	1332	*Burkholderia gladioli* BSR3 ǀ 83% ǀ 96%	*Burkholderia*
908	2956	4290	+	1335	-	Unassigned
938	11318	12652	-	1335	*Variovorax* sp. HW608 ǀ 85% ǀ 96%	*Variovorax*
1516	6842	8140	-	1299	*Achromobacter denitrificans* PR1 ǀ 99% ǀ 100%	*Achromobacter*
1823	7634	8041	-	408	*Bordetella hinzii* NCTC13200 ǀ 83% ǀ 87%	*Bordetella*

3847	97	1416	+	1320	*Cupriavidus taiwanensis* LMG19424 | 96% | 99%	*Cupriavidus*
7226	1140	2117	-	978	*Pseudomonas oryzae* KCTC 32247 | 78% | 86%	*Pseudomonas*
9750	972	1433	-	462	*Legionella hackeliae* 798-PA-H | 81% | 96%*	*Legionella*
12169	50	169	+	120	*Stenotrophomonas maltophilia* NCTC10257 | 96% | 100%	*Stenotrophomonas*
15096	23	370	+	348	*Stenotrophomonas acidaminiphila* ZAC14D2 | 93% | 100%	*Stenotrophomonas*
25559	94	396	+	303	*Chryseobacterium* sp. 36-9 | 100% | 100%*	*Chryseobacterium*
28434	23	667	+	645	*Rhodospirillum centenum* SW | 83% | 97%	*Rhodospirillum*
58142	32	487	-	456	*Variovorax boronicumulans* J1 | 95% | 100%	*Variovorax*
					**Total**	**19**
**Ring-hydroxylating dioxygenases**						
	**Anthranilate / Ortho-halobenzoate 1,2-dioxygenase (EC 1.14.12.1)**					
10	248828	250222	-	1395	*Pseudomonas aeruginosa* W45909 | 99% | 100%	*Pseudomonas*
331	4188	5498	-	1311	*Bordetella bronchiseptica* I328 | 83% | 97%	*Bordetella*
614	9931	11118	-	1188	*Pseudomonas aeruginosa* PP4 | 99% | 91%	*Pseudomonas*
999	87	257	+	171	*Hydrogenophaga* sp. T4 | 86% | 78%*	*Hydrogenophaga*
1974	6226	7485	-	1260	*Cupriavidus* sp. USMAA2-4 | 75% | 95%	*Cupriavidus*
2429	1892	3145	-	1254	*Hydrogenophaga* sp. PBC | 78% | 91%	*Hydrogenophaga*
3141	1827	3134	+	1308	*Pigmentiphaga* sp. NDS-2 | 97% | 100%	*Pigmentiphaga*
4439	1931	3226	+	1296	*Cupriavidus basilensis* 4G11 | 85% | 90%	*Cupriavidus*
6331	818	2056	-	1239	-	Unassigned
18612	792	962	-	171	*Variovorax paradoxus* B4 | 85% | 100%	*Variovorax*
33891	65	604	+	540	-	Unassigned
					**Total**	**11**
	**Benzoate 1,2-dioxygenase (EC 1.14.12.10)**					
35	196596	197720	+	1125	*Pseudomonas resinovorans* NBRC 106553 | 82% | 97%	*Pseudomonas*
130	64866	65900	+	1035	-	Unassigned
1342	20	346	+	327	*Cupriavidus* sp. NH9 | 98% | 100%	*Cupriavidus*
9867	132	1358	-	1227	*Sinorhizobium* sp. RAC02 | 87% | 98%	*Sinorhizobium*
6	11347	12738	+	1392	*Pseudomonas resinovorans* NBRC 106553 | 89% | 98%	*Pseudomonas*
645	10636	11979	-	1344	*Bordetella* sp. SCN 67-23 | 96% | 99%*	*Bordetella*
929	3123	4445	-	1323	*Bordetella* sp. SCN 67-23 | 98% | 99%*	*Bordetella*
2832	2663	3859	+	1197	*Bordetella* sp. SCN 67-23 | 96% | 99%*	*Bordetella*
3622	6	1094	+	1089	*Bordetella* sp. SCN 67-23 | 98% | 100%*	*Bordetella*
9022	373	1587	-	1215	*Bordetella* sp. SCN 67-23 | 96% | 99%*	*Bordetella*
1052	4824	5099	-	276	*Parvibaculum lavamentivorans* DS-1 | 85% 64%*	*Parvibaculum*
1124	5319	6737	+	1419	*Sphingobium* sp. PNB | 100% | 100%	*Sphingobium*
1382	5661	6908	-	1248	*Bordetella* sp. SCN 67-23 | 97% | 100%*	*Bordetella*
1824	2551	3888	-	1338	*Sphingomonas* sp. DN1 | 99% | 100%	*Sphingomonas*
4332	44	949	+	906	-	Unassigned
					**Total**	**15**
	**Naphthalene 1,2-dioxygenase (EC 1.14.12.12)**					
73	110789	112057	+	1269	*Sphingomonas wittichii* RW1 | 79% | 94%	*Sphingomonas*
466	20834	22153	+	1320	*Sphingomonas flavimaris* SMR4y | 88% | 100%	*Sphingomonas*
493	7477	8664	+	1188	*Sphingomonas* sp. LH128 | 100% | 100%	*Sphingomonas*
890	235	1581	-	1347	*Sphingomonas* sp. P2 | 100% | 100%	*Spingomonas*
1164	2	520	+	519	*Bordetella* sp. SCN 67-23 | 98% | 100%*	*Bordetella*
1565	4776	6128	+	1353	*Sphingomonas* sp. Km187 | 100% | 100%	*Sphingomonas*
1565	6674	8041	+	1368	*Sphingobium* sp. PNB | 100% | 100%	*Sphingobium*
3181	2872	4236	+	1365	*Sphingobium yanoikuyae* B1 | 100% | 100%	*Sphingobium*
9595	75	953	+	879	*Bordetella* sp. SCN 67-23 | 96% | 100%*	*Bordetella*
					**Total**	**9**
	**2-halobenzoate 1,2-dioxygenase (EC 1.14.12.13)**					
313	29097	30452	+	1356	*Sphingobium* sp. TKS | 90% | 100%	*Sphingobium*
497	12138	13484	+	1347	*Achromobacter denitrificans* PR1 | 99% | 100%	*Achromobacter*
810	8207	9538	-	1332	*Achromobacter* sp. MFA1 R4 | 77% | 91%	*Achromobacter*
2567	5693	5971	-	279	*Novosphingobium resinovorum* SA1 | 88% | 86%	*Novosphingobium*
12681	8	907	+	900	*Bordetella* sp. SCN 67-23 | 97% | 98%*	*Bordetella*
48945	329	538	-	210	*Variovorax boronicumulans* J1 | 93% | 95%	*Variovorax*
					**Total**	**6**
	**Biphenyl 2,3-dioxygenase (EC 1.14.12.18)**					
493	16568	16978	+	411	*Novosphingobium aromaticivorans* DSM 12444 | 99% | 100%	*Novosphingobium*
2111	3684	4115	-	432	*Novosphingobium* sp. PP1Y | 98% | 96%	*Novosphingobium*
9656	847	1257	+	411	*Comamonas testosteroni* P19 | 89% | 100%	*Comamonas*
89	74526	75800	-	1275	*Pseudomonas pseudoalcaliigenes* KF707 | 95% | 100%	*Pseudomonas*
1124	8215	9495	+	1281	*Sphingobium* sp. PNB | 100% | 100%	*Sphingobium*
1824	6034	7302	+	1269	*Sphingomonas* sp. DN1 | 100% | 100%	*Sphingomonas*
					**Total**	**6**
	**3-phenylpropanoate dioxygenase (EC 1.14.12.19)**					
281	18169	19488	-	1320	*Achromobacter denitrificans* PR1 | 99% | 100%	*Achromobacter*
598	12742	14088	-	1347	*Achromobacter denitrificans* PR1 | 99% | 100%	*Achromobacter*
666	15671	16831	-	1161	*Achromobacter xylosoxidans* MN001 | 99% | 100%	*Achromobacter*
					**Total**	**3**
	**p-cumate 2,3-dioxygenase (EC 1.14.12.25)**					
30	172310	173620	-	1311	*Paraburkholderia xenovorans* LB400 | 79% | 90%	*Paraburkholderia*
49	115435	116709	+	1275	*Pseudomonas aeruginosa* W60856 | 100% | 100%	*Pseudomonas*
131	7034	8308	+	1275	-	Unassigned
					**Total**	**3**

	**Other/Putative/Unclassified ring-hydroxylating dioxygenases**					
45	85972	87132	-	1161	*Pseudomonas resinovorans* NBRC 106553 ǀ 84% ǀ 96%	*Pseudomonas*
125	39321	40610	+	1290	*Sphingobium* sp. RAC03 ǀ 75% ǀ 99%	*Sphingobium*
216	48582	49055	-	474	*Acidovorax avenae* ATCC 19860 ǀ 83% ǀ 95%	*Acidovorax*
220	44610	45680	-	1071	*Sphingobium herbicidovorans* HM ǀ 80% ǀ 99%	*Sphingobium*
267	32963	34300	-	1338	*Novosphingobium resinovorum* SA1 ǀ 88% ǀ 99%	*Novosphingobium*
422	10966	12327	-	1362	*Achromobacter denitrificans* PR1 ǀ 99% ǀ 100%	*Achromobacter*
425	1443	2912	+	1470	*Novosphingobium* sp. AP12 ǀ 94% ǀ 98%*	*Novosphingobium*
490	15279	16445	-	1167	*Novosphingobium* sp. AP12 ǀ 93% ǀ 100%*	*Novosphingobium*
500	10911	12266	-	1356	*Sphingomonas* sp. S5-249 ǀ 76% ǀ 98%*	*Sphingomonas*
602	11513	12823	-	1311	*Variovorax* sp. Root473 ǀ 81% ǀ 100%*	*Variovorax*
642	13369	14799	+	1431	*Novosphingobium resinovorum* SA1 ǀ 88% ǀ 98%	*Novosphingobium*
1039	3758	4933	+	1176	*Rhodoferax* sp. DCY110 ǀ 85% ǀ 95%	*Rhodoferax*
1246	5545	6534	-	990	-	Unassigned
1613	6046	7422	+	1377	*Novosphingobium resinovorum* SA1 ǀ 84% ǀ 99%	*Novosphingobium*
1656	1746	2972	-	1227	-	Unassigned
2067	1896	2999	-	1104	*Cupriavidus nantongensis* X1 ǀ 97% ǀ 100%	*Cupriavidus*
2472	445	1782	-	1338	*Sphingobium* sp. RAC03 ǀ 84% ǀ98%	*Sphingobium*
2593	1674	2825	+	1152	*Parvibaculum lamentivorans* DS-1 ǀ 99% ǀ 99%*	*Parvibaculum*
3751	2511	3572	+	1062	*Bordetella* sp. SCN 67-23 ǀ 95% ǀ 100%*	*Bordetella*
4409	2037	3437	-	1401	-	Unassigned
4723	2040	3107	-	1068	*Sphingobium* sp. MI1205 ǀ 80% ǀ 96%	*Sphingobium*
7358	23	346	+	324	*Acidovorax* sp. KKS102 ǀ 93% ǀ 90%	*Acidovorax*
8191	1465	1890	-	426	*Novosphingobium* sp. AP12 ǀ 95% ǀ 88%*	*Novosphingobium*
10438	706	1410	-	705	-	Unassigned
11469	829	1281	-	453	*Achromobacter xylosoxidans* NH44784-1996 ǀ 85% ǀ 93%	*Achromobacter*
11804	810	1271	-	462	*Bordetella* sp. SCN 67-23 ǀ 96% ǀ 100%*	*Bordetella*
45485	20	205	+	186	*Labrys* sp. WJW ǀ 97% ǀ 100%*	*Labrys*
30504	76	624	+	549	-	Unassigned
8205	1599	1844	-	246	-	Unassigned
1124	2042	3304	-	1263	*Sphingobium* sp. PNB ǀ 99% ǀ 100%	*Sphingobium*
					**Total**	**30**

*Blastp was used instead blastn for genus assignation, as blastn did not show any significant hits.
